# Design, synthesis and evaluation of benzodioxole and bromofuran tethered 1,2,4-triazole hybrids as potential anti breast cancer agents with computational insights

**DOI:** 10.1038/s41598-025-09420-1

**Published:** 2025-07-16

**Authors:** Manjunath R, Sushruta S. Hakkimane, Shashikala B S, Santhosh L. Gaonkar

**Affiliations:** 1 Department of Chemistry, Manipal Institute of Technology, Manipal Academy of Higher Education, Manipal, Karnataka 576104 India; 2 Department of Biotechnology, Manipal Institute of Technology Bengaluru, Manipal Academy of Higher Education, Manipal, Karnataka 576104 India; 3 Department of Physics, Sri Jayachamarajendra College of Engineering, JSS Technical Institutions Campus, JSS Science and Technology University, Mysuru, Karnataka 570006 India

**Keywords:** Benzo[*d*] [1,3] dioxole, 5-Bromofuran, 3,4-(Methylenedioxy) phenylacetic acid, 1,2,4-Triazoles, DFT, MEP, Lipinski’s rule of five, Jorgensen’s rule of three, Biochemistry, Cancer, Computational biology and bioinformatics, Drug discovery, Chemistry

## Abstract

1,2,4-Triazole derivatives are the focus of extensive research in medicinal chemistry because of their diverse biological activities, particularly their potential as anticancer agents. In this study, we designed and synthesized six compounds featuring benzo[*d*][1,3]dioxole and 5-bromofuran tethered to 1,2,4-triazole hybrids. This was achieved through an efficient four-step protocol with reproducible results. The characterization of the 1,2,4-triazoles was conducted via various analytical techniques, including FTIR, ^1^H NMR, ^13^C NMR, and mass spectrometry. The in vitro anti-breast cancer activities of synthesized 1,2,4-triazols were evaluated against the MCF-7 cell line using the MTT assay. Among all the synthesized compounds, compound **12b** demonstrated an excellent IC_50_ value of 3.54 ± 0.265 µg/mL. Additionally, in silico studies, such as molecular docking to predict the orientation of the compounds, molecular dynamics simulations to evaluate binding stability with the target protein, drug-likeness studies to evaluate Lipinski’s rule of five and Jorgensen’s rule of three, DFT analysis to determine the energy gap of the frontier molecular orbitals (FMOs) and the molecular electrostatic potential (MEP) for identifying sites of nucleophilic and electrophilic attacks, were also conducted.

**Supplementary Information:**

The online version contains supplementary material available at 10.1038/s41598-025-09420-1.

## Introduction

Every year, approximately 1.8 million women across the globe are diagnosed with breast cancer, making it the most common cancer among females^1^. Breast cancer has several subtypes of diverse neoplasms. These subtypes are grouped into four categories according to the immunohistochemical expression of hormone receptors, such as progesterone receptor-positive (PR+), estrogen receptor-positive (ER+), human epidermal growth factor receptor-positive (HER2+), and triple-negative breast cancer (TNBC), which are characterized by the absence of expression of any of the above receptors^2^. TNBC is characterized by the absence of both HER2 and hormone receptors^3,4^. Targeting these hormone receptors is the primary strategy for treating breast cancer.

Despite significant advances in treatment, chemotherapy continues to be the primary approach for managing breast cancer. However, it is worrying that more than 90% of patients with metastatic breast cancer experience treatment failure. The prevalence of chemotherapy resistance is particularly alarming, impacting at least 25% of patients and underscoring the urgent need for more effective treatment strategies and further research to address chemotherapeutic resistance^5–8^.

The mechanisms of resistance in breast cancer are intricate and not yet fully understood. Cancer cells can develop resistance to certain classes of cytotoxic treatments because of modifications in target proteins and cellular biological processes. Key alterations include increased DNA damage repair, decreased drug metabolism, reduced membrane permeability, impaired apoptosis, and increased energy-dependent efflux of hydrophobic drugs^9–11^.

Current chemotherapeutic agents, such as doxorubicin, paclitaxel, cisplatin, thiotepa, and chlorambucil, have proven ineffective in halting the proliferation of resistant cancer cells^12^. This drug resistance arises primarily because cancer cells adapt to the unique structures and mechanisms of drugs, which lack selectivity for tumor cells^13^. Additionally, the cytotoxic and genotoxic properties of these chemotherapeutics can damage healthy soft tissues, further complicating cancer treatment^14^. As a result, a new generation of more versatile chemotherapeutic agents is urgently needed.

**Fig. 1:**
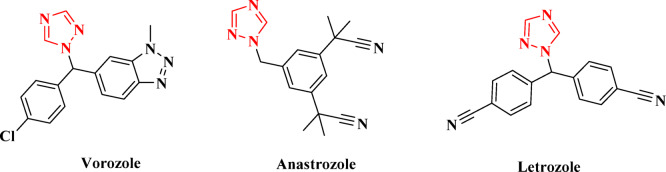
Anticancer drugs having a 1,2,4-triazole moiety.

Ongoing research in anticancer drug development aims to discover new treatments that offer reduced toxicity and increased efficacy. The five-membered heterocyclic ring known as the triazole and its derivatives serve as effective and versatile chemotherapeutic agents. Triazoles are categorized into two types based on the nitrogen atom’s position within the heterocyclic ring: 1,2,3-triazoles and 1,2,4-triazoles. Among these structural isomers, 1,2,4-triazoles are the most significant anticancer agents. This is evidenced by several commercially available anticancer drugs, such as letrozole, vorozole, and anastrozole, which feature a 1,2,4-triazole scaffold in their molecular structures^15,16^, as illustrated in Fig. 1.

**Fig. 2:**
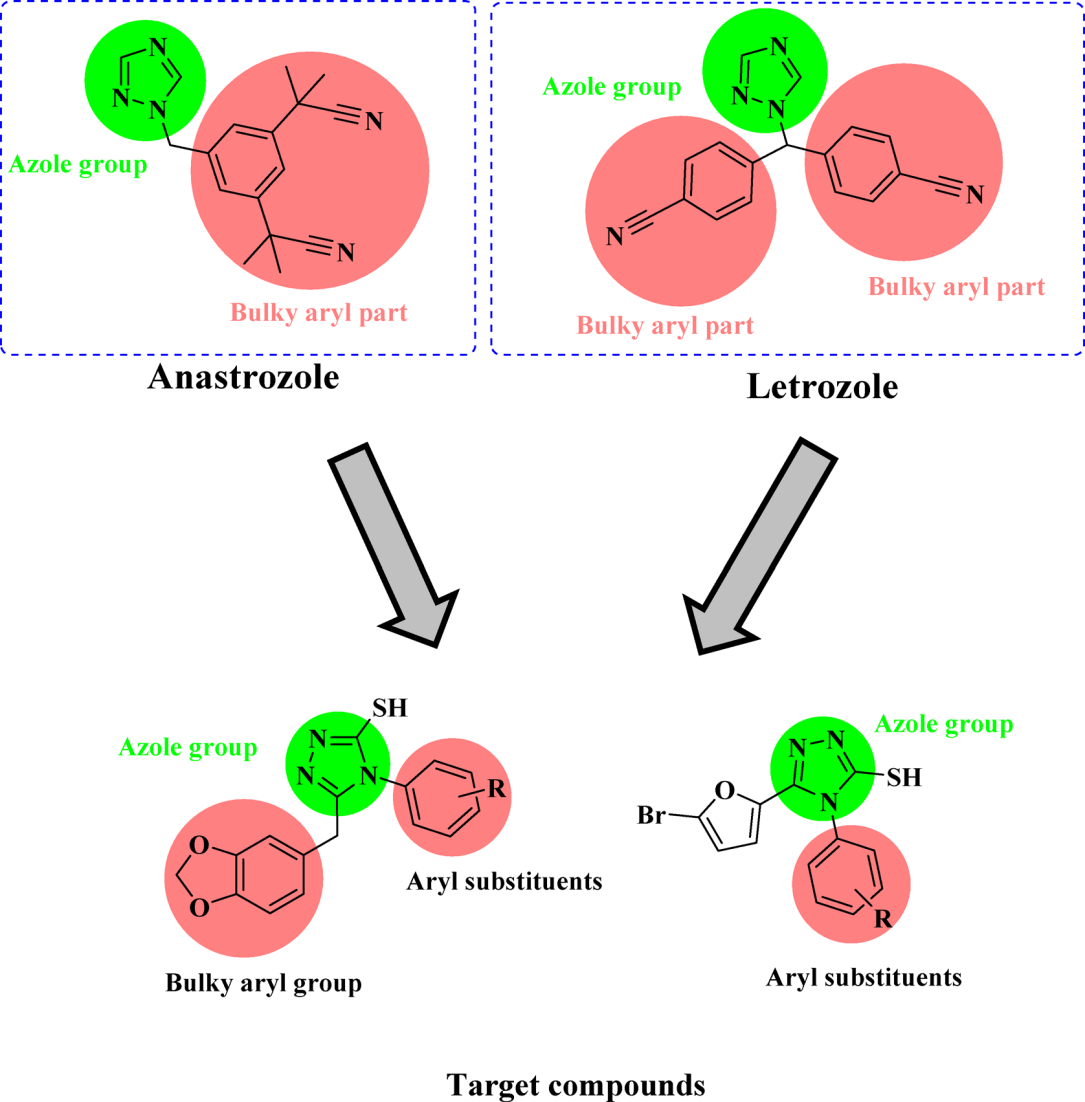
Design of 1,2,4-triazole hybrids.

A key strategy in drug discovery involves integrating two or more pharmacophoric moieties into a single molecular entity. By combining various pharmacophores, researchers can create novel molecules that interact with multiple biological targets. This multifaceted approach enhances both the potency and efficacy of the compounds, enabling the exploration of unique mechanisms of action that may not be attainable with individual pharmacophoric entities. In this context, 1,2,4-triazole hybrids with benzo[*d*] [1,3] dioxole and 5-bromofuran moieties were designed (Fig. 2) and synthesized via an isothiocyanate-mediated reaction^17–19^. The anticancer properties of these synthesized triazole hybrids were carefully evaluated against the MCF-7 breast cancer cell line^20^.

## Results and discussion

###  Chemistry

**Scheme 1:**
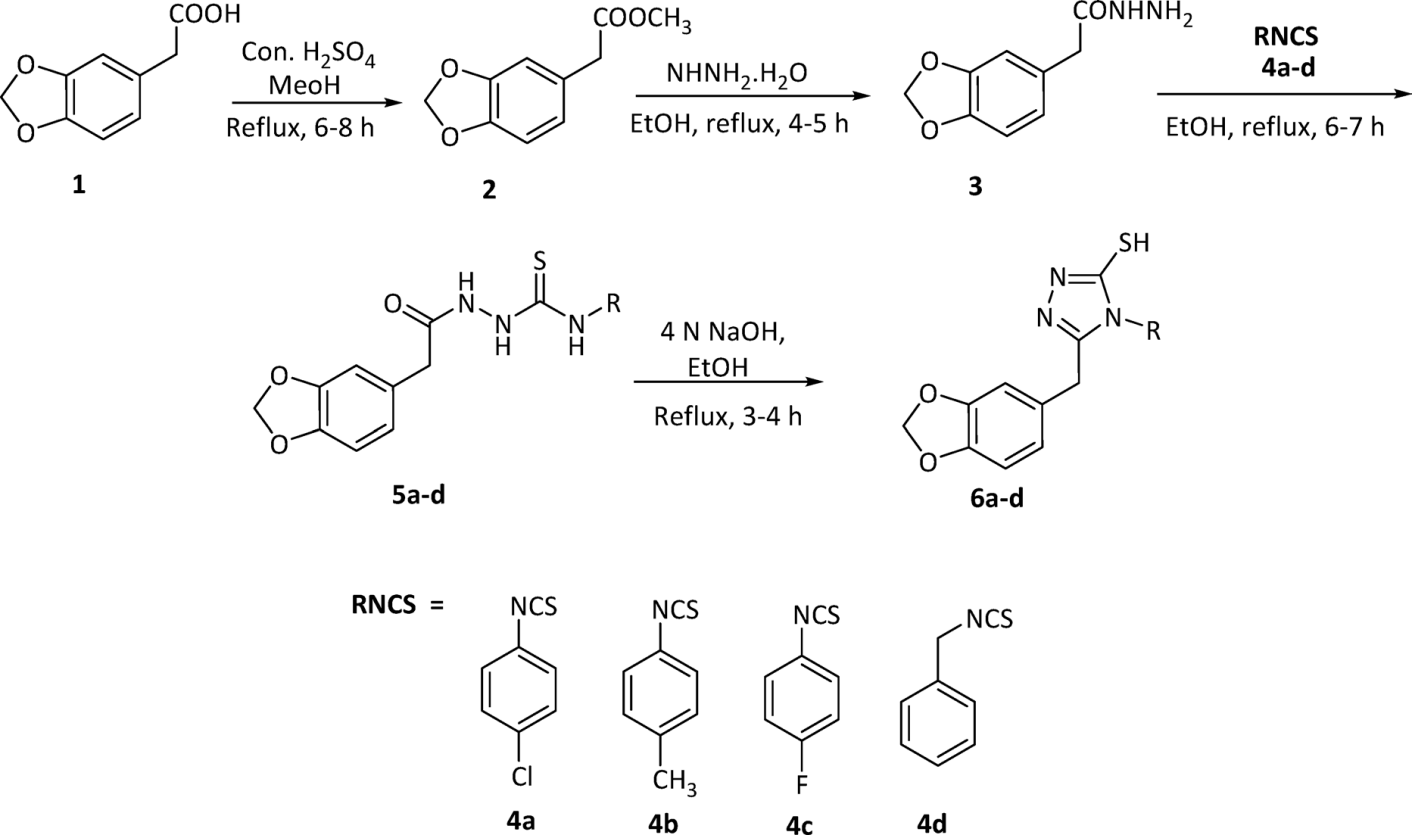
Synthesis of benzo[*d*] [1,3] dioxole-tethered 1,2,4-triazole hybrids.

**Scheme 2:**
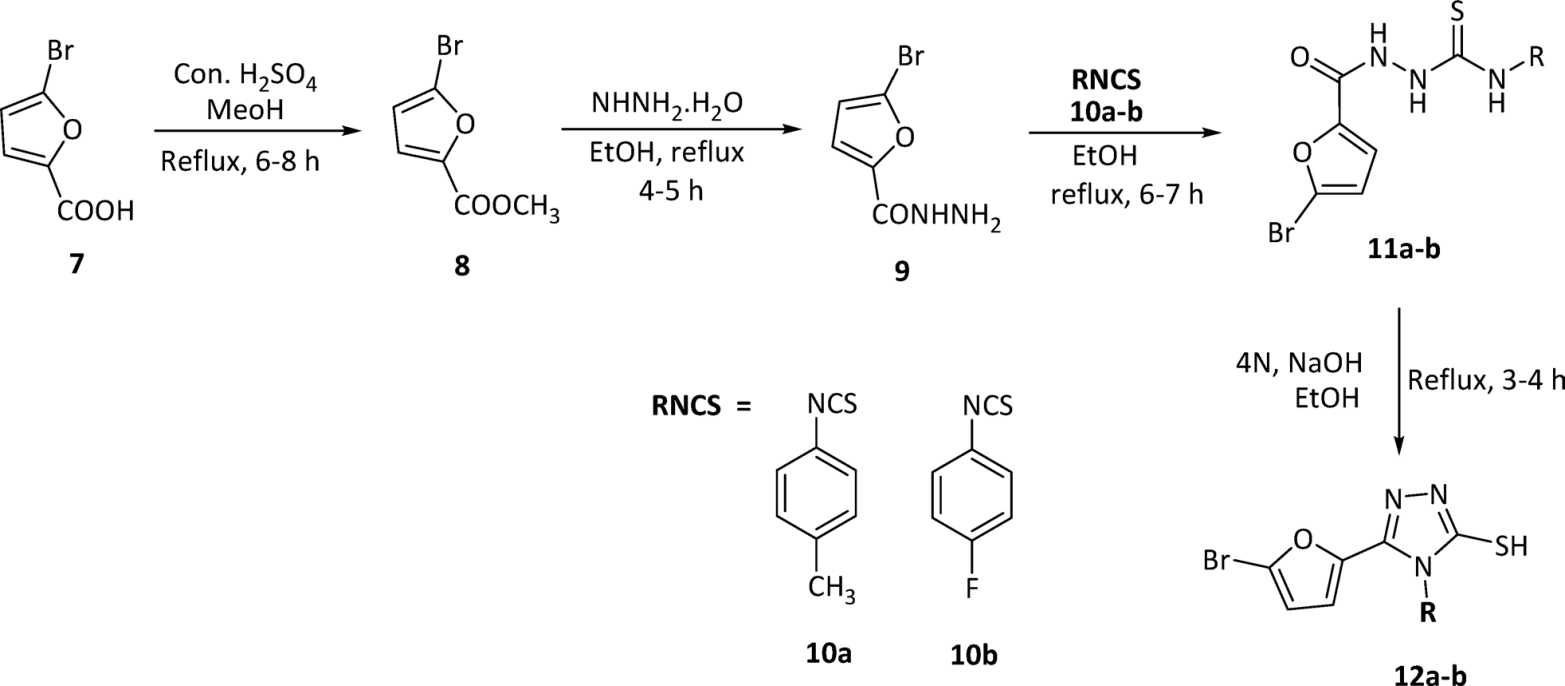
Synthesis of 5-bromofuran-tethered 1,2,4-triazole hybrids.

In the present study, six 1,2,4-triazole hybrids were synthesized, as illustrated in Schemes 1 and 2. The process begins with the esterification of 3,4-(methylenedioxy)phenylacetic acid and 5-bromofuranoic acid, forming the corresponding esters. These esters then reacted with hydrazine hydrate, leading to the formation of hydrazides **3** and **9**. Intermediates **5a-d** and **11a-b** were obtained by reacting hydrazides **3** and **9** with various phenyl isothiocyanates (RNCS). Ultimately, the formation of 1,2,4-triazole hybrids (**6a-d** and **12a-b**) occurred through the cyclization of intermediates **5a-d** (as depicted in Scheme 1) and **11a-b** (as shown in Scheme 2) in the presence of 4 N sodium hydroxide.

**Scheme 3:**
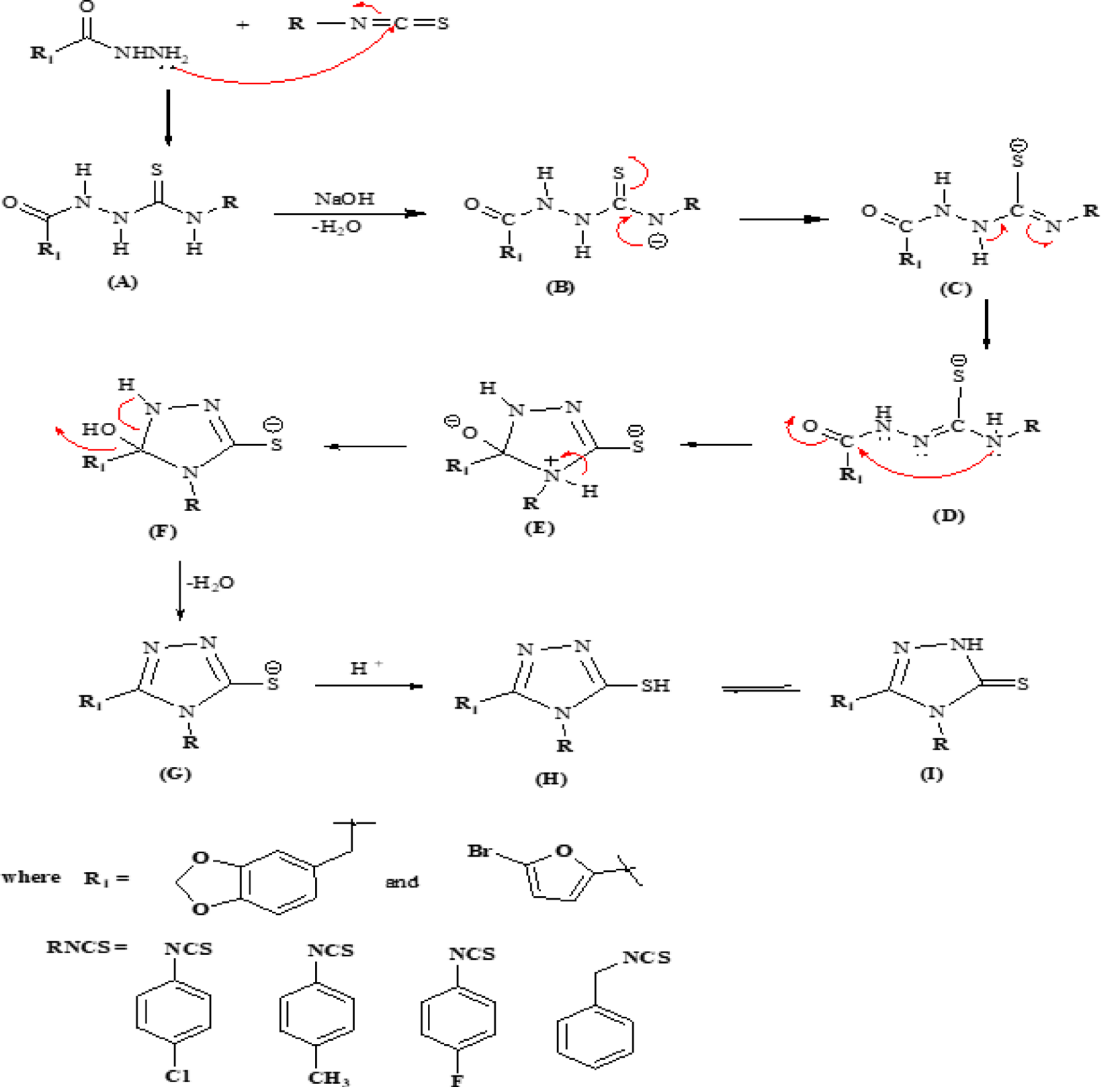
Plausible mechanism of benzo[*d*][1,3]dioxole and 5-bromofuran-tethered 1,2,4-triazole formation.

A plausible mechanism for the formation of 1,2,4-triazoles is illustrated in Scheme 3. Acid hydrazide reacts with isothiocyanate, the nitrogen atom of the hydrazine group attacks the carbon atom of the isothiocyanate, leading to the formation of a thiosemicarbazide (A). The base abstracts a proton from thiosemicarbazide (A) with the formation of a thioamide intermediate (B), which further undergoes proton shift to produce the thiolate intermediate (C). In the next step, the nucleophile attacks the carbonyl carbon (D), leading to ring closure and the creation of a 5-membered intermediate (E). Following this, proton transfer occurs alongside the elimination of water (F), and subsequent protonation (G) results in the formation of 1,2,4-triazoles (H). The final product undergoes tautomerization between the thiol (H) and thione (I) forms, which is characteristic of 1,2,4-triazoles.

The synthesized 1,2,4-triazoles were characterized through spectral studies, including FTIR, ^1^H NMR, ^13^C NMR, and mass spectrometry. The FTIR data for 1,2,4-triazoles (compounds **6a-d** and **12a-b**) exhibited distinctive bands at specific wavenumbers, such as 561 (C–Br), 778 (C–Cl), 1600 (C=N), 1499 (C=C), 2752 (SH), 1219 (C–F), 3074 (aromatic C–H), and 2947 (aliphatic C–H) cm^− 1^. The ^1^H NMR spectra of these triazoles revealed characteristic signals at δ 3.7 and δ 5.9, corresponding to methylene groups, as well as signals at δ 7.2 and 7.5, attributed to aromatic protons, and a δ 13.8 signal was representative of the thiol group. Additionally, compound **6b** exhibited a resonance at δ 2.3, indicating a methyl functional group, whereas compound **12a** displayed signals at δ 6.8 and 7.2 for protons in the furan ring, with an additional signal at δ 2.1 for another methyl group. The ^13^C NMR spectrum showed distinct chemical shifts for the carbon atoms in the 1,2,4-triazole ring, with notable signals observed at approximately 151 and 168 ppm, confirming the successful formation of the 1,2,4-triazole. Furthermore, signals corresponding to the benzo[*d*][1,3]dioxole carbons were identified at approximately 146 and 147 ppm, whereas the carbon atoms in the furan ring appeared at approximately 114 and 120 ppm. The mass peaks for compounds **6a** and **12b** were recorded at m/z values of 346.19 and 340.11, respectively.

###  In vitro anticancer activity

The MTT assay is a widely accepted technique for measuring metabolic activity in live cells, using non-treated controls and cell-free blanks for comparison. The in vitro anticancer potential of synthesized 1,2,4-triazole derivatives (**6a-d** and **12a-b**) was assessed against the MCF-7 cell line. Cells were propagated in humidified conditions, maintaining 5% CO_2_ and 37 °C temperature. The IC_50_ values of the triazole derivatives were compared with those of standard doxorubicin, as detailed in Table 1. The derivatives derived from benzo [*d*] [1,3] dioxole **(6a-d)** presented IC_50_ values ranging from 7.989 ± 1.65 to 22.03 ± 2.33 µg/mL against MCF-7 cells, with compounds **6a** and **6b** showing good inhibition. In contrast, the 5-bromofuran-derived triazoles (**12a** and **12b)** demonstrated inhibitory potentials of 12.8 ± 0.645 µg/mL and 3.54 ± 0.265 µg/mL, respectively, compared with the standard drug doxorubicin, which had an IC_50_ value of 0.5785 ± 0.0095 µg/mL against MCF-7 cells.

**Fig. 3:**
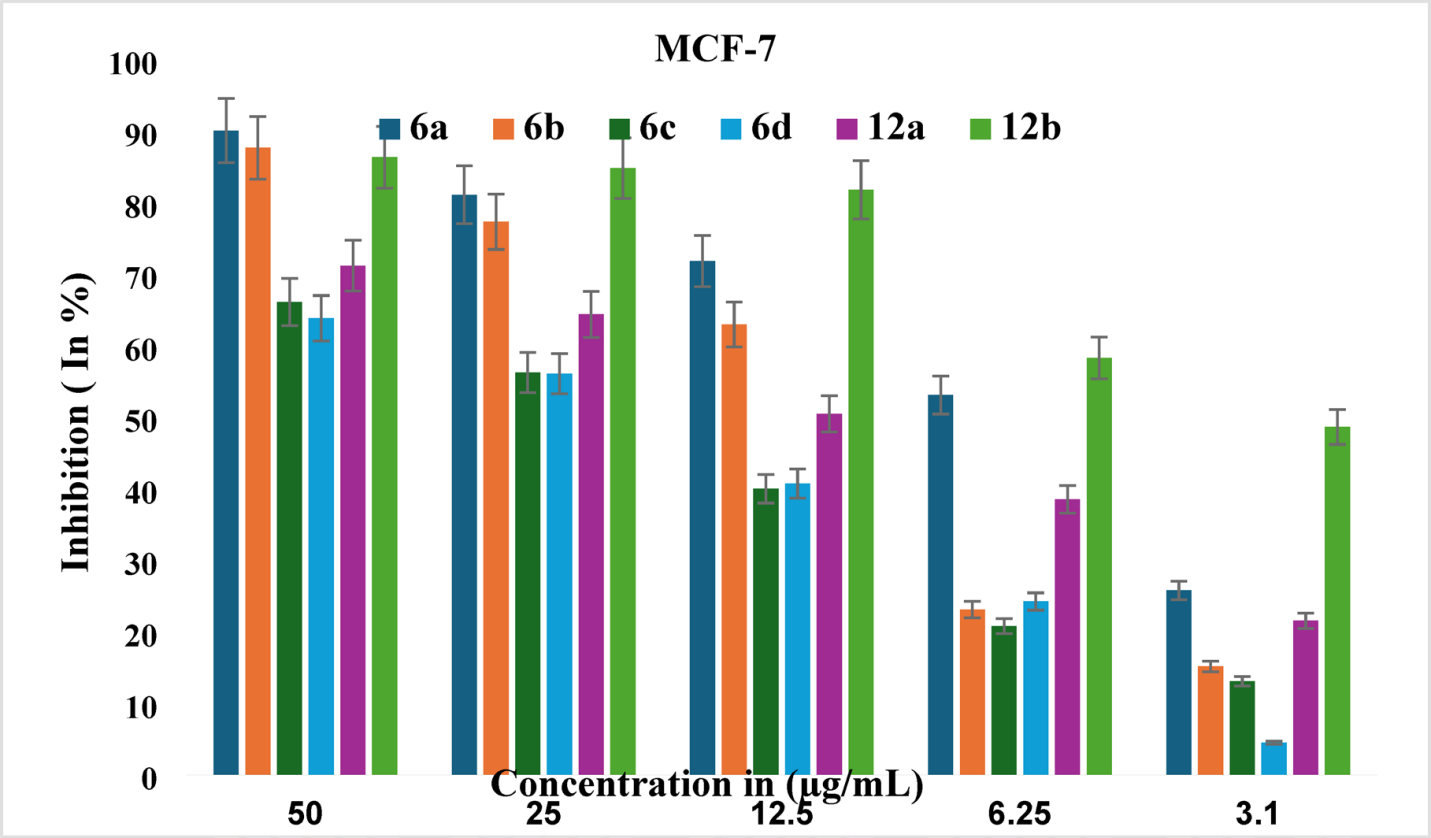
Percentage inhibition of compounds **6a-d** and **12a-b**.

An examination of the inhibitory profiles of the triazole derivatives (**6a-d** and **12a-b**) against MCF-7 cells revealed that compound **12b** exhibited the most favourable inhibitory profile, with an IC_50_ value of 3.54 ± 0.265 µg/mL. This is followed by compound **6a**, which has an IC_50_ value of 7.989 ± 1.65 µg/mL. A bar graph representing percentage inhibition at various concentrations of triazole derivatives (**6a-d** and **12a-b**) is shown in Fig. 3.

**Table 1:** IC_50_ values of the 1,2,4-triazole hybrids against MCF-7 cells.

Sl.no	Compound	MCF-7 (µg/mL)
1	6a	7.989 ± 1.65
2	6b	15.8 ± 2.26
3	6c	20.62 ± 0.415
4	6d	22.03 ± 2.33
5	12a	12.8 ± 0.645
6	12b	3.54 ± 0.265
7	Doxorubicin	0.5785 ± 0.0095

### Computational studies

#### Molecular docking

To understand intricate ligand‒protein binding interactions, in silico molecular docking studies were carried out *via* the Glide module of Schrödinger 2024 − 3^21^. For this purpose, the protein was sourced from the Protein Data Bank through the RCSB PDB repository. This enabled us to investigate the binding affinities and orientations of the ligands within the active sites of the protein.

For docking studies focused on anticancer evaluation, we selected human estrogen receptor alpha (PDB ID: 3ERT) in complex with 4-hydroxytamoxifen^22^. The choice of protein is based on the fact that estrogen receptor alpha is a crucial target in hormone-responsive breast cancers, such as MCF-7^23,24^.

**Fig. 4:**
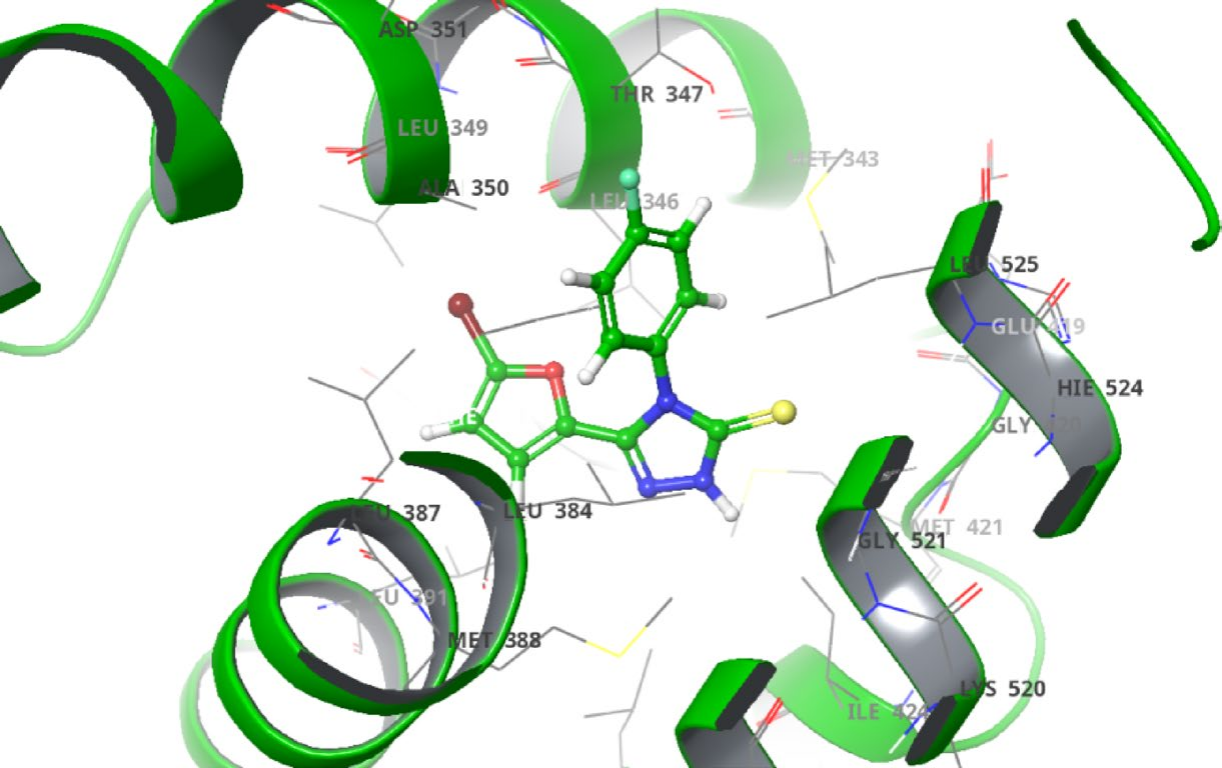
3D docking pose of compound **12b** inside the binding pocket.

**Fig. 5:**
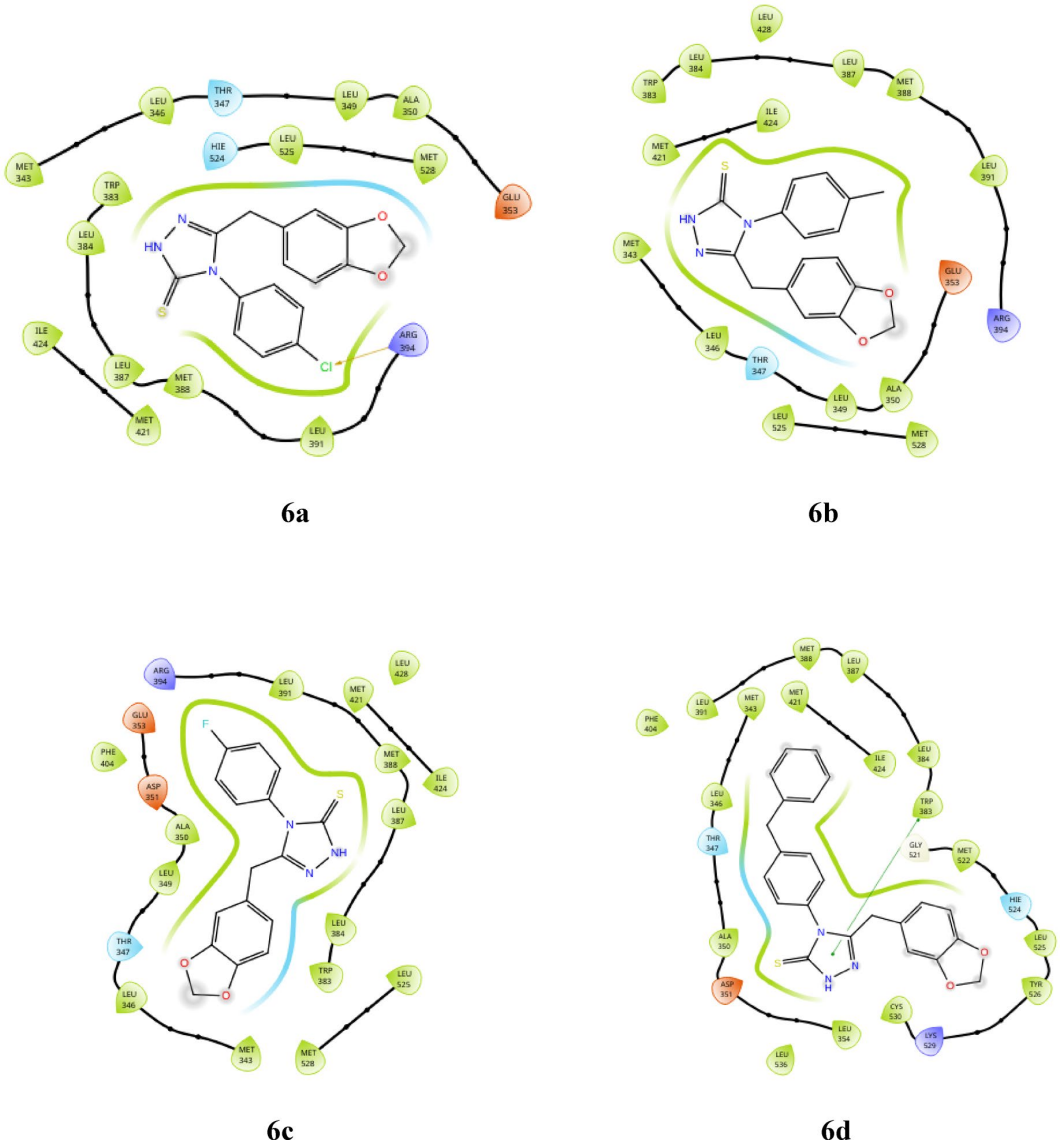

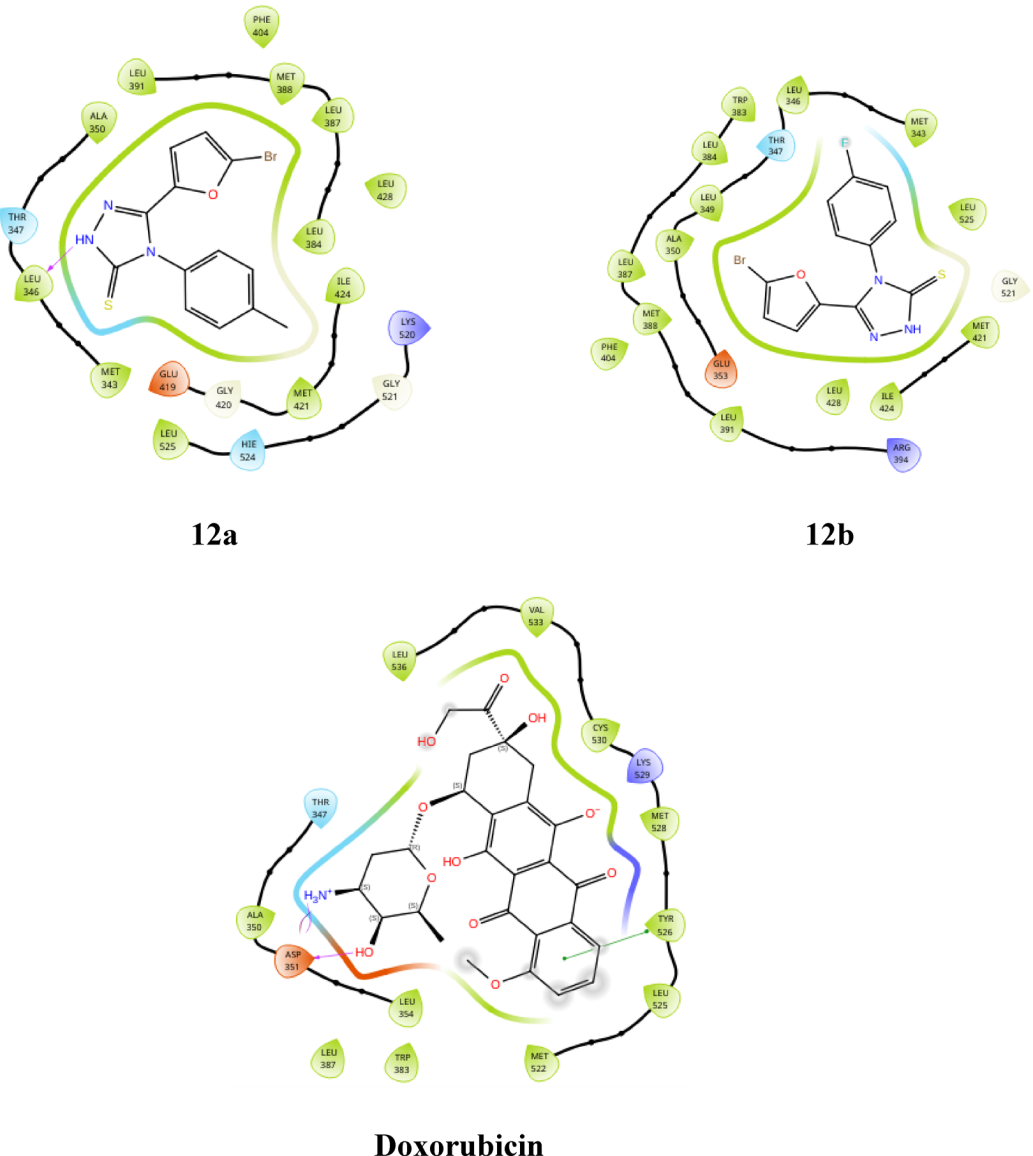
2D docking poses of compounds **6a-d**, **12a-b** and doxorubicin.

The derivatives **(6a-d** and **12a-b)** exhibited hydrophobic interactions with various amino acids, including LEU 391, MET 398, LEU 387, LEU 384, TRP 383, ALA 350, LEU 349, MET 343, LEU 346, THR 347, HIE 524, and MET 398. Additionally, compound **6a** demonstrated halogen bonding interactions with ARG 394. These compounds also displayed polar interactions with THR 347 and HIE 524 and an aromatic hydrogen bond with LEU 346, THR 347, and THR 526. Furthermore, compound **6d** showed Pi-Pi stacking with TRP 383, and compound **12a** exhibited a hydrogen bond interaction with LEU 346. The detailed interactions are presented in Table 2. Docking analysis revealed compound **12b** has a docking score of -7.845 kcal/mol, which is notably lower (more negative) than standard doxorubicin, which was recorded as -4.846 kcal/mol. This finding indicates that compound **12b** is the most promising candidate among the derivatives. Additionally, compound **12b** achieved the highest glide score of -8.229 kcal/mol among the triazole derivatives, underscoring its superior binding affinity for the target protein compared with that of doxorubicin. In terms of the glide energy, compound **6d** has the highest value of -44.083 kcal/mol, whereas compound **12b** has a moderate glide energy of -41.974 kcal/mol, and compound **12a** has the lowest glide energy of -15.731 kcal/mol. It is noteworthy that compound **6d** has a lower docking and glide score than **12b**, but it has a higher glide energy. This discrepancy can be attributed to favorable interactions offset by steric clashes, desolvation penalties, and ligand strain in compound **12b**. This imbalance is particularly significant for compound **12a**, which accounts for its lower glide energy. The docking scores, glide scores, glide energies, and potential interactions of compounds **6a-d** and **12a-b** are detailed in Table 2. The 3D docking pose of compound **12b** is illustrated in Fig. 4 (remaining are provided in supplementary file), whereas the 2d poses for compounds **6a-d**, **12a-b**, and doxorubicin are shown in Fig. 5.

**Table 2:** Docking score, glide score, glide energy, and various interactions of the compounds **(6a-d** and **12a-b)**.

Sl. No	Compound name	Docking score	Glide score	Glide energy	Interactions
1	**6a**	− 5.724	− 7.278	− 43.779	*Hydrophobic*: ALA 350, LEU 349, LEU 346, MET 343, TRP 383, LEU 384, LEU 387, MET 388, LEU 391, LEU 525
					*Halogen*: ARG 394 (2.30 Å) *Aromatic H-bond* : LEU 346 (2.57 Å), THR 347 (2.64 Å)
2	**6b**	− 5.822	− 7.376	− 37.498	*Hydrophobic*: MET 343, LEU 346, LEU 349, ALA 350, LEU 391, MET 388, LEU 387, LEU 384, TRP 383
					*Aromatic H-bond*: LEU 346 (2.70 Å)
3	**6c**	− 6.244	− 7.798	− 41.032	*Hydrophobic*: TRP 383, LEU 384, LEU 387, MET 388, LEU 391, ALA 350, LEU 349
					*Aromatic H-bond*: LEU 387 (2.37 Å), THR 347 (2.30 Å)
4	**6d**	− 6.308	− 7.862	− 44.083	*Hydrophobic*: LEU 346, MET 343, CYS 530, TYR 526, LEU 525, LEU 354, ALA 350
					*Aromatic H-bond*: THR 347 (2.39 Å), THR 526 (3.64 Å)
					*Pi-Pi stacking*: TRP 383 (5.07 Å)
5	**12a**	− 7.746	− 8.201	− 15.731	*Hydrophobic*: MET 343, ALA 350, LEU 391, MET 388, ALA 350, LEU 391, MET 388, LEU 387, LEU 384, PHE 404, ILE 424, LEU 428
					*H-bond*: LEU 346 (1.82 Å)
**6**	**12b**	− 7.845	− 8.299	− 41.974	*Hydrophobic*: MET 343, LEU 346, TRP 383, LEU 384, LEU 349, LEU 387, MET 388, ALA 350, LEU 391, LEU 428, ILE 424, MET 421, LEU 525
7	Doxorubicin	− 4.846	− 6.667	− 38.244	*H-bond*: ASP 351 (1.98 and 1.79 Å)
					*Pi-Pi stacking*: TYR 526 (3.47 Å)
					*Salt-bridge*: ASP 351 (4.45 Å)
					*Hydrophobic*: LEU 536, TRP 383, LEU 354, LEU 387, ALA 350, MET 522, LEU 525, TYR 526, MET 528, CYS 530, VAL 533,

####  MD simulation

**Fig. 6:**
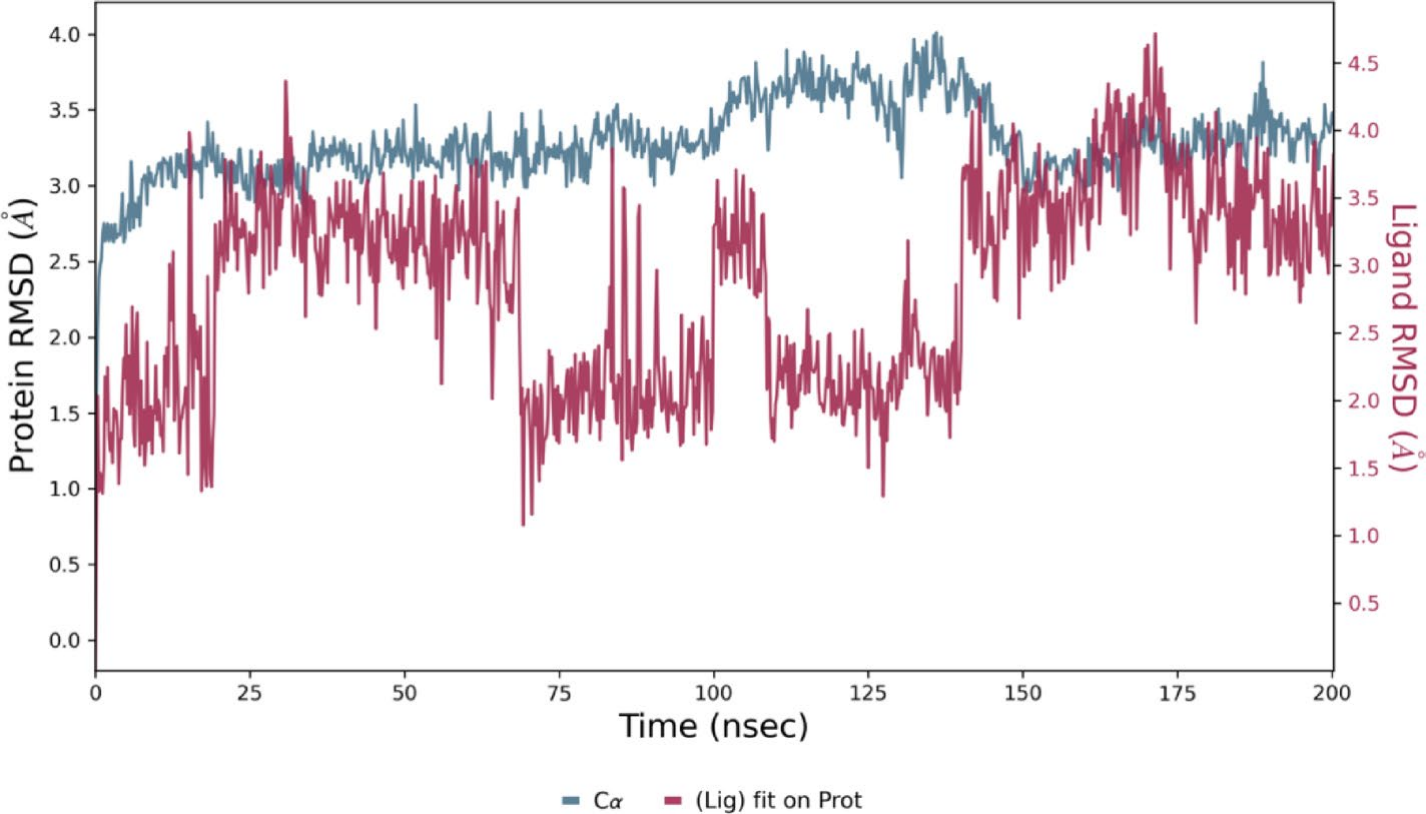
RMSD graph of compound **12b** for a 200 ns run.

The stability of the binding conformation of docked structure **12b** (IC_50_: 3.54 ± 0.265 µg/mL) was evaluated through molecular dynamics simulations using the Desmond module of Schrödinger 2024 − 3^25^. As illustrated in Fig. 6, the simulations were conducted for 200 ns to assess the binding stability. The resulting simulated trajectories were analysed by calculating the RMSD values, highlighting the binding complex’s robustness in relation to the protein backbone. The ligand RMSD ranges from 1.5 to 4.5 Å, while the protein RMSD falls between 2.0 and 4.0 Å. The protein-ligand RMSD did not reach stability until 25 ns, after this point, up to 75 ns, it almost stabilised, exhibiting minimal fluctuations. Between 75 and 150 ns, the protein-ligand RMSD showed variability within acceptable limits of 1 to 3 Å, indicating no significant changes in protein conformation or ligand diffusion from the binding pocket. After 150 ns, the protein-ligand RMSD reached equilibrium. Compound **12b** displayed the least fluctuations in protein-ligand RMSD throughout the 200 ns simulation period, suggesting a stable binding interaction. This stability was further corroborated by MM-GBSA analysis, which revealed a favourable binding free energy of -58.5364 kcal/mol for compound **12b**, reflecting strong and effective interactions with the target protein. Given these findings, compound **12b** is a promising candidate for future research due to its stability and high affinity. These results align with the structural characteristics that enhance binding efficiency.

**Fig. 7:**
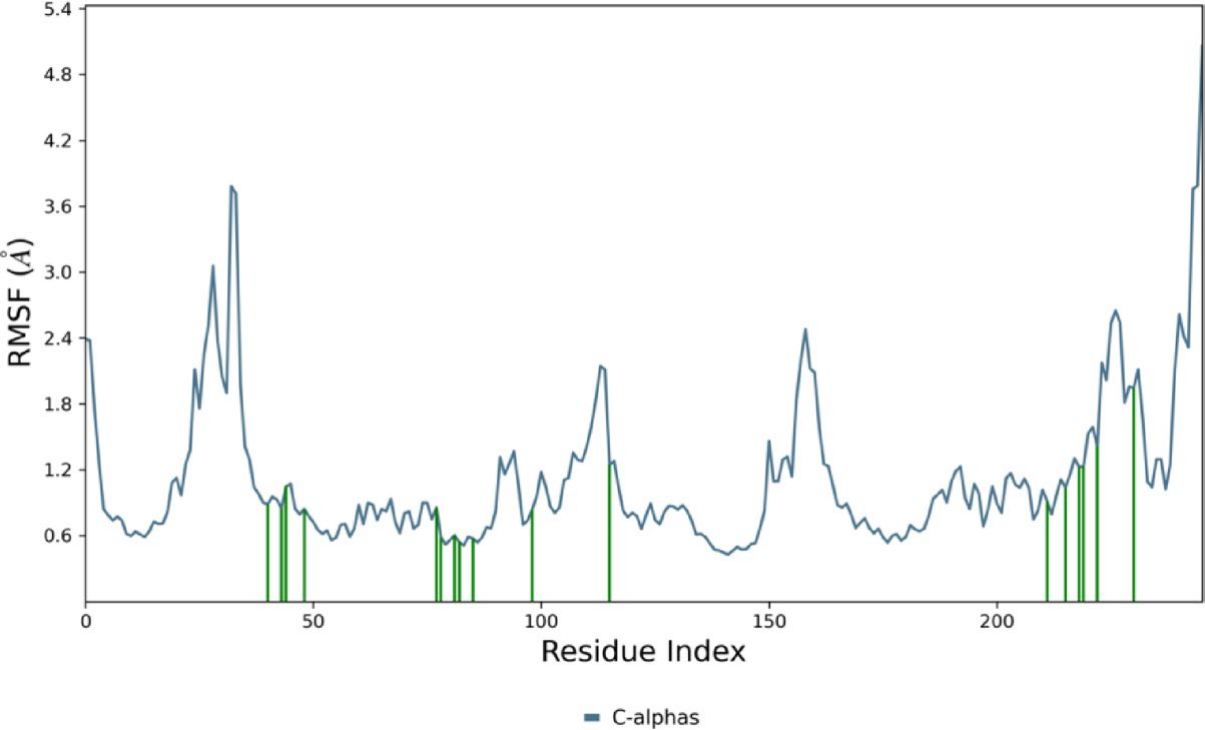
Protein RMSF of compound **12b**.

A root mean square fluctuation (RMSF) analysis was carried out to evaluate the flexibility and mobility of different amino acids concerning compound **12b**, as shown in Fig. 7. The findings indicate that a higher RMSF value is associated with increased flexibility. In contrast, a lower RMSF value signifies greater rigidity in the amino acid residues, and a green dash highlights the flexibility and mobility of amino acid residues in contact with compound **12b.**

**Fig. 8:**
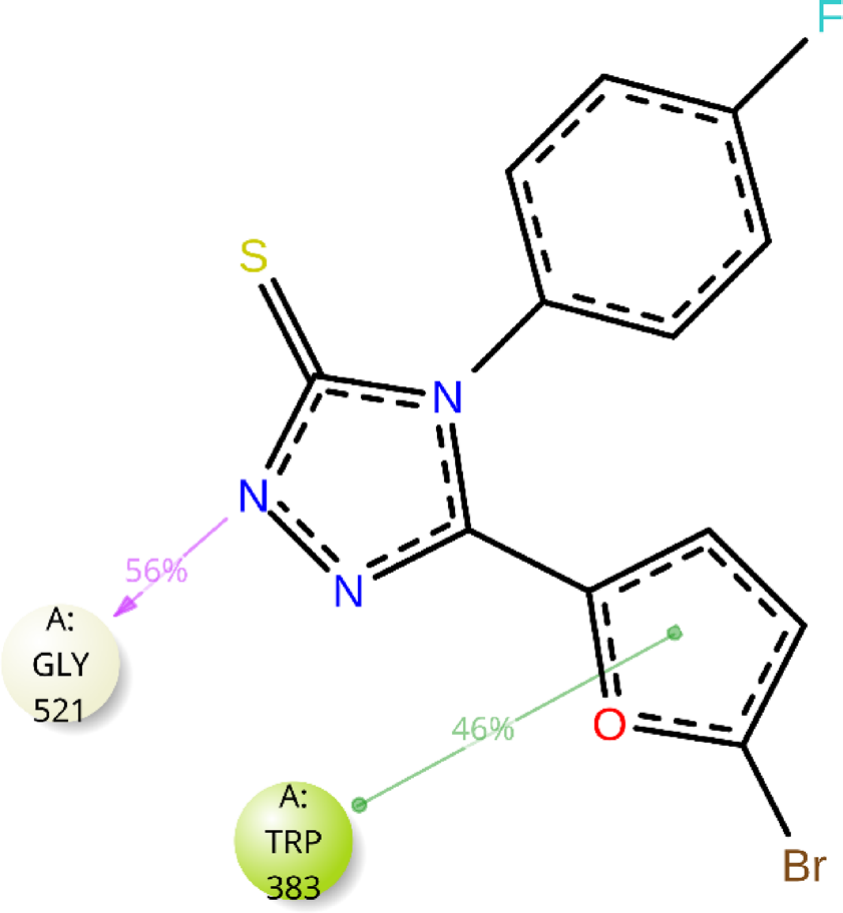
Graphical depiction of the ligand‒atom contacts of compound **12b** with amino acid residues of the protein.

**Fig. 9:**
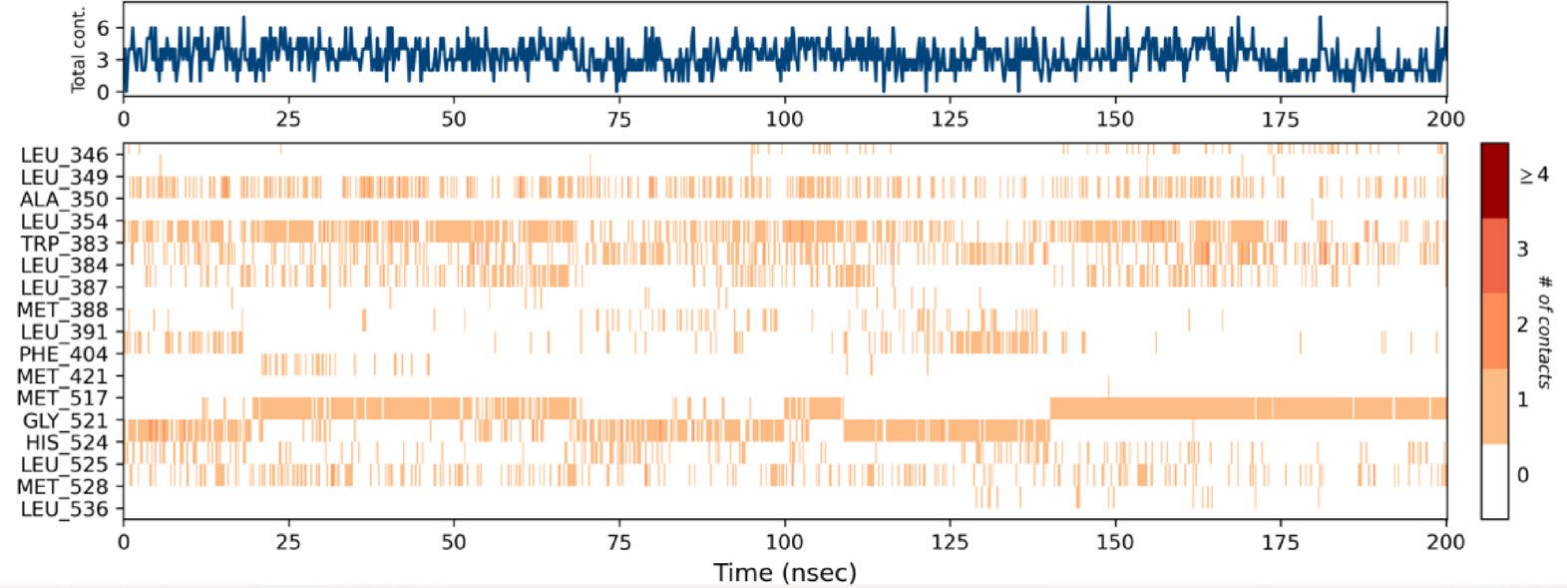
Timeline representation showing the binding interactions of compound **12b** with amino acid residues over a simulation time of 200 ns.

**Fig. 10:**
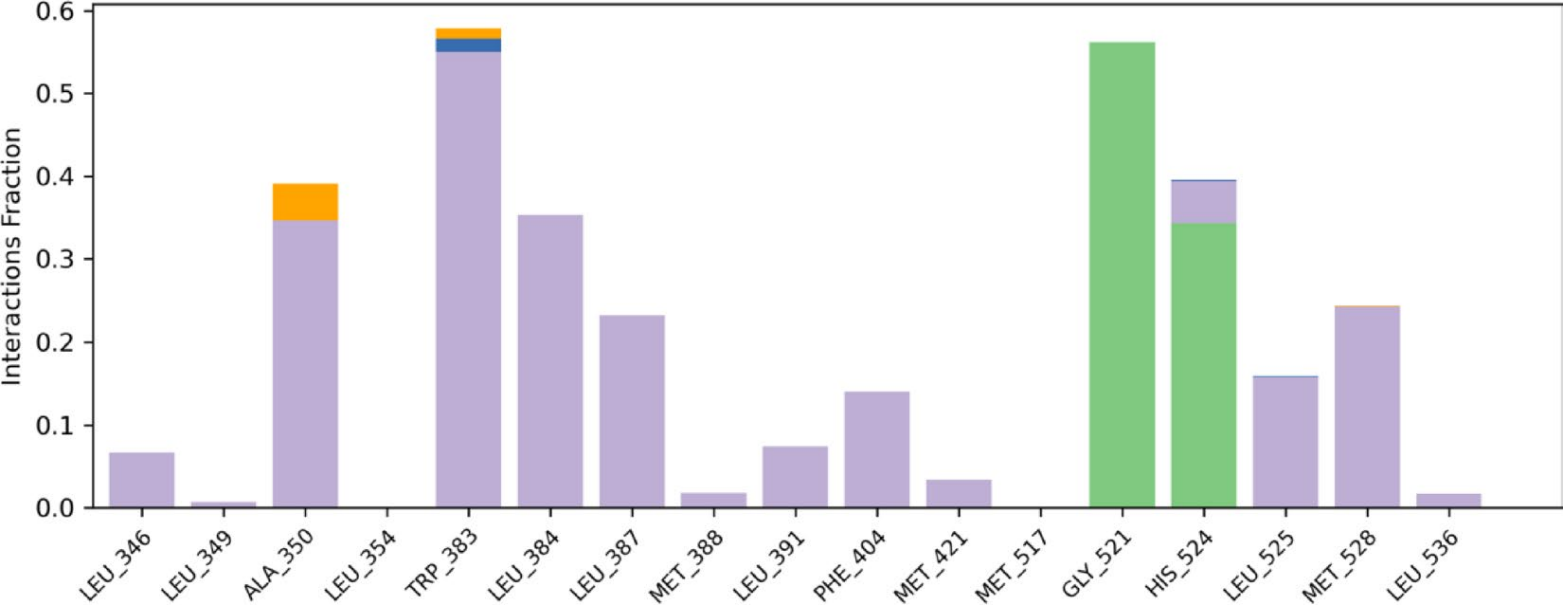
Graphical depiction of the protein‒ligand contact histogram of compound **12b**.

The interactions between the ligand atoms of compound **12b** and the amino acid residues of the protein are illustrated in Fig. 8. Notably, the amino acid TRP 383 (46%) interacts with the furan ring via pi-pi stacking, and GLY 521 (56%) interacts with the nitrogen atom of the triazole ring via hydrogen bonding. Analysis of the timeline depicted in Fig. 9 reveals that the amino acids GLY 521 and TRP 383 consistently engaged with compound **12b** throughout each trajectory. Moreover, compound **12b** exhibited hydrogen bond interactions of 55% with GLY 521 and 35% with HIS 524. It also displayed halogen bond interactions of 40% with ALA 350 and 55% with TRP 383. Additionally, significant hydrophobic interactions were observed with TRP 383 (55%), LEU 384 (35%), ALA 350 (35%), and HIS 524 (40%), as illustrated in Fig. 10. These interactions are anticipated to enhance the inhibitory potential of compound **12b**.

**Fig. 11:**
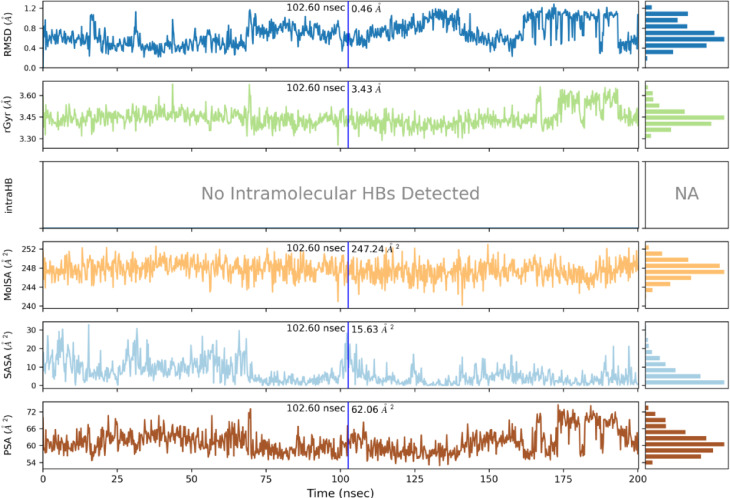
Ligand properties of compound **12b**.

Ligand properties, such as the ligand RMSD, radius of gyration (rGyr), intramolecular hydrogen bonds (intra HBs), molecular surface area (MoISA), solvent accessible area (SASA), and polar surface area (PSA) of compound **12b**, were calculated and are presented in Fig. 11. The results indicate that compound **12b**, with a ligand RMSD of 0.46 Å, is extremely stable inside the binding site and has a radius of gyration of 3.43 Å, indicating a compact and rigid molecular structure throughout the simulation. No intramolecular hydrogen bonds were observed, suggesting that the conformational stability and binding affinity primarily arise from interactions with the protein’s binding site, which is further supported by the low ligand RMSD and favourable MM-GBSA value. Compound **12b** has a molecular surface area of 247.24 Å², indicative of a compact structure, likely a snug fit with moderate solvent exposure and interactions occurring in a buried environment. The solvent accessible area was measured at 15.63 Å², reflecting limited solvent exposure and strong hydrophobic interactions. Furthermore, the polar surface area of 62.06 Å² suggests balanced polarity and lipophilicity.

#### Drug-Likeness prediction via Qikprop

The QikProp module in Schrödinger 2024-3 was used to evaluate drug likeness^26^. Table 3. Shows the drug likeliness of compounds **6a-d** and **12a-b.**

In drug discovery, drug-likeness is a crucial parameter that determines the essential physicochemical and pharmacokinetic properties required for a compound to be a successful drug. Derivatives, **6a-d** and **12a-b**, adhere to Lipinski’s rule of five, indicating a favourable balance of molecular weight, lipophilicity, and hydrophilicity (as assessed by the octanol/water partition coefficient, QplogPo/W), and the number of hydrogen bond acceptors and donors, with no violations. Furthermore, Jorgensen’s rule of three has been applied to these derivatives, revealing that Caco-2 permeability exceeds 22 nm/sec, while active metabolites register below 7. It is vital to note that derivatives conform to QPlogS (predicted aqueous solubility), they exhibit 100% projected human oral absorption and exceptional Caco-2 permeability. They also align with Lipinski’s rule of five. This indicates that under optimal physiological conditions, the derivatives possess favourable qualities for oral bioavailability. Furthermore, the derivatives comply with QPlogPC16 (hexadecane/gas partition coefficient), QPlogPoct (octanol/gas partition coefficient), QPlogKhsa (binding to human serum albumin), and Polar surface area (PSA), highlighting favourable lipophilicity, membrane permeability, and serum albumin binding. This indicates that these compounds are likely capable of crossing biological membranes.

Compound **12b** has demonstrated a CNS value of + 2, indicating a significant potential for central nervous system activity, mainly due to lipophilic fluorine and bromine groups. However, it is crucial to highlight that these computational estimates are derived solely from physicochemical properties, and they need in vivo studies to confirm any CNS effects.

**Table 3:** Drug-likeness of compounds **6a-d** and **12a-b**.

Compound	Lipinski’s rule of five					QPlogPC16	QPlogPoct
	Molecular weight	Donor hydrogen bond	Acceptor Hydrogen bond	QplogPo/W	_*n*_ _violations_		
Range	< 500	≤ 5	≤ 10	≤ 5	≤ 1	4–18	8–43
**6a**	345.80	1	5	3.6	0	10.5	15.5
**6b**	325.39	1	5	3.5	0	10.2	15.4
**6c**	329.35	1	5	3.4	0	9.6	15.1
**6d**	325.39	1	5	4.3	0	12.04	17.9
**12a**	334.97	1	4	3.3	0	9.5	14.3
**12b**	338.95	1	4	3.3	0	9.0	14.0

Compound	% Human oral absorption	Qplog Khsa	CNS	PSA	Jorgensen’s rule of three		
					QPPCaco	Primary metabolites	QPlogS
Range	> 80% is high, < 25% is poor	− 1.5 to 1.5	− 2 (inactive), + 2 (active)	7–200	> 22 nm/sec	< 7	> − 5.7
**6a**	100	0.196	1	59.77	2984.80	1	-4.7
**6b**	100	0.246	1	59.62	2802.03	2	-4.6
**6c**	100	0.129	1	59.71	2991.83	1	-4.3
**6d**	100	0.54	0	60.58	2327.14	2	-4.5
**12a**	100	0.245	1	47.1	2465.98	2	-4.7
**12b**	100	0.133	2	47.13	2462.22	1	-4.6

####  DFT analysis

The molecular geometry and type of substituents attached to the derivatives under study are often associated with their stability and reactivity^27,28^. Hence, a DFT study was carried out using the Jaguar module of Schrödinger 2024-3 with B3LYP-D_3_ for theory and 6-31Gss for the basis set in the gas phase^29^.

Frontier molecular orbitals (FMOs) consist of the lowest unoccupied molecular orbital (LUMO) and the highest occupied molecular orbital (HOMO). The LUMO, being unoccupied and having the lowest energy, can accept electrons and function as an electron acceptor. In contrast, the HOMO is occupied by electrons and possesses the highest energy level, making it an electron donor. The interaction between ligands and binding sites is predominantly influenced by frontier molecular orbitals^30^. Among the 1,2,4-triazoles derived from benzo[*d*][1,3]dioxole **(6a-d)**, compound **6a** displays the lowest energy difference (∆E) of 4.62 eV, whereas compound **6d** has a higher ∆E of 5.142 eV. The remaining compounds, **6b** and **6c**, have moderate ∆E values of 4.973 eV and 4.821 eV, respectively. In the case of the bromo furan-derived 1,2,4-triazoles, compounds **12a** and **12b** have energy difference values of 4.617 eV and 4.608 eV, respectively.

In summary, compound **12b** exhibited a smaller energy gap of 4.608 eV, suggesting higher reactivity. This reduced energy gap typically implies that a compound can more readily participate in chemical reactions. In contrast, compound **6d** demonstrated a larger energy gap of 5.142 eV, which indicates greater stability. A wider energy gap often correlates with a compound’s resistance to chemical changes, making it less reactive. Overall, these energy gap values highlight a clear distinction in the reactivity and stability profiles of the compounds.

The physicochemical properties, such as electronegativity, hardness, softness, electrophilicity, and nucleophilicity, of compounds **6a-d** and **12a-b** were calculated and are summarized in Table 4. These findings indicate compound **12b** has the highest electronegativity, reflecting a strong ability to attract electrons and moderate hardness. This combination suggests an effective balance between stability and reactivity. Furthermore, compound **12b** displays moderate softness, increased electrophilicity, and minimal nucleophilicity, making it a promising candidate for further drug development studies.

**Fig. 12:**
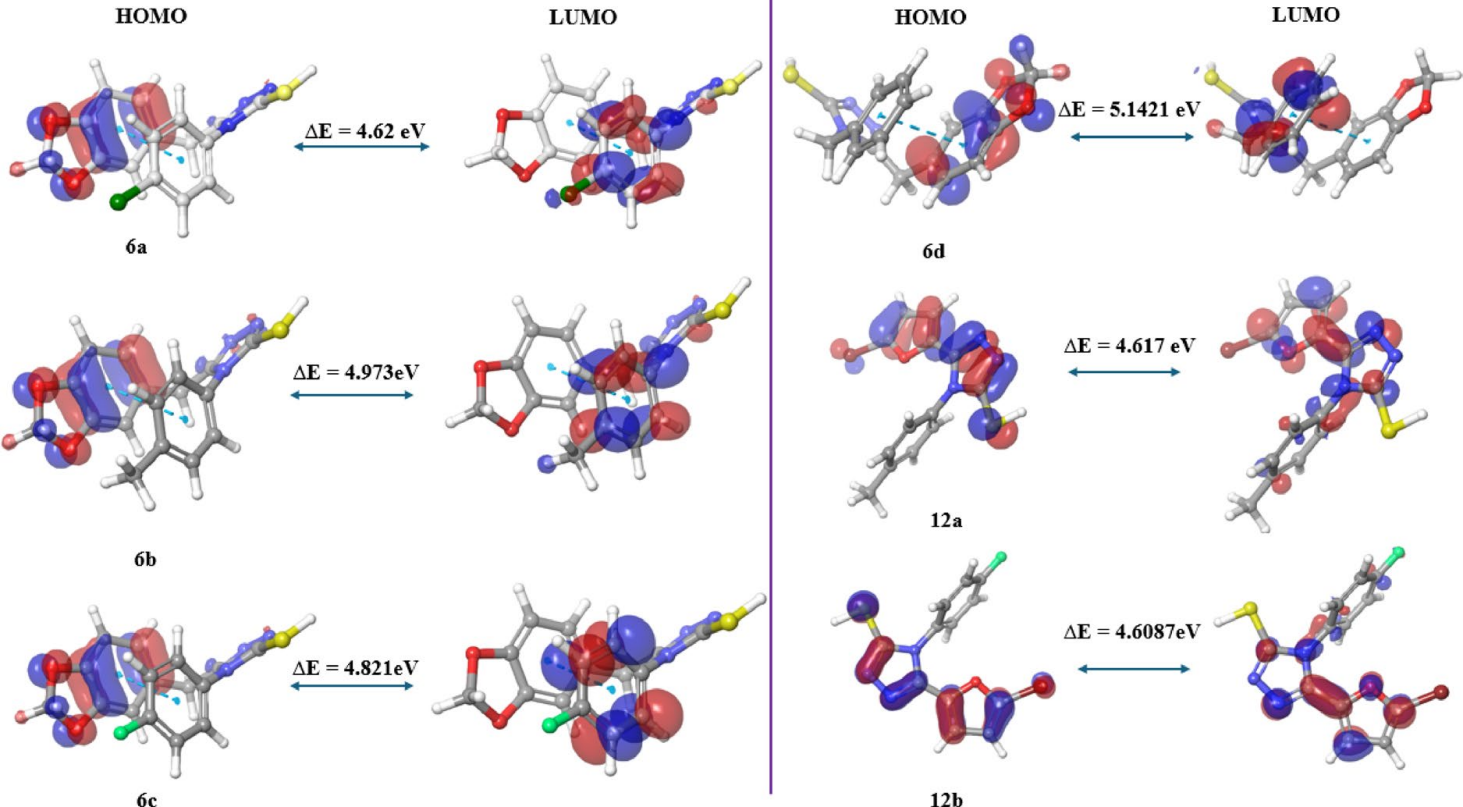
HOMO, LUMO, and energy gap (∆E) of compounds **6a-d** and **12a-b** in the gas phase.

The HOMO of compounds **6a-d** is attributed to the benzo[*d*][1,3]dioxole moiety, whereas the LUMO comprises aromatic substituents. Similarly, for compounds **12a** and **12b**, the HOMO is associated with the Bromo furan moiety, with the LUMO again consisting of aromatic substituents. The HOMO and LUMO energy levels for the compounds investigated in the gas phase, along with their energy gaps (∆E), are presented in Fig. 12.

**Table 4:** Theoretical calculations of compounds **6a-d** and **12a-b**.

Compound name	Electronegativity (χ)	Hardness (η)	Softness (σ)	Electrophilicity (ω)	Nucleophilicity (ε)
**6a**	3.452	2.310	0.432	1.289	0.775
**6b**	3.220	2.486	0.402	1.042	0.959
**6c**	3.362	2.410	0.4148	1.172	0.852
**6d**	3.225	2.510	0.398	1.035	0.965
**12a**	3.342	2.281	0.438	1.223	0.817
**12b**	3.475	2.304	0.434	1.310	0.763

####  Molecular electrostatic potential analysis

**Fig. 13:**
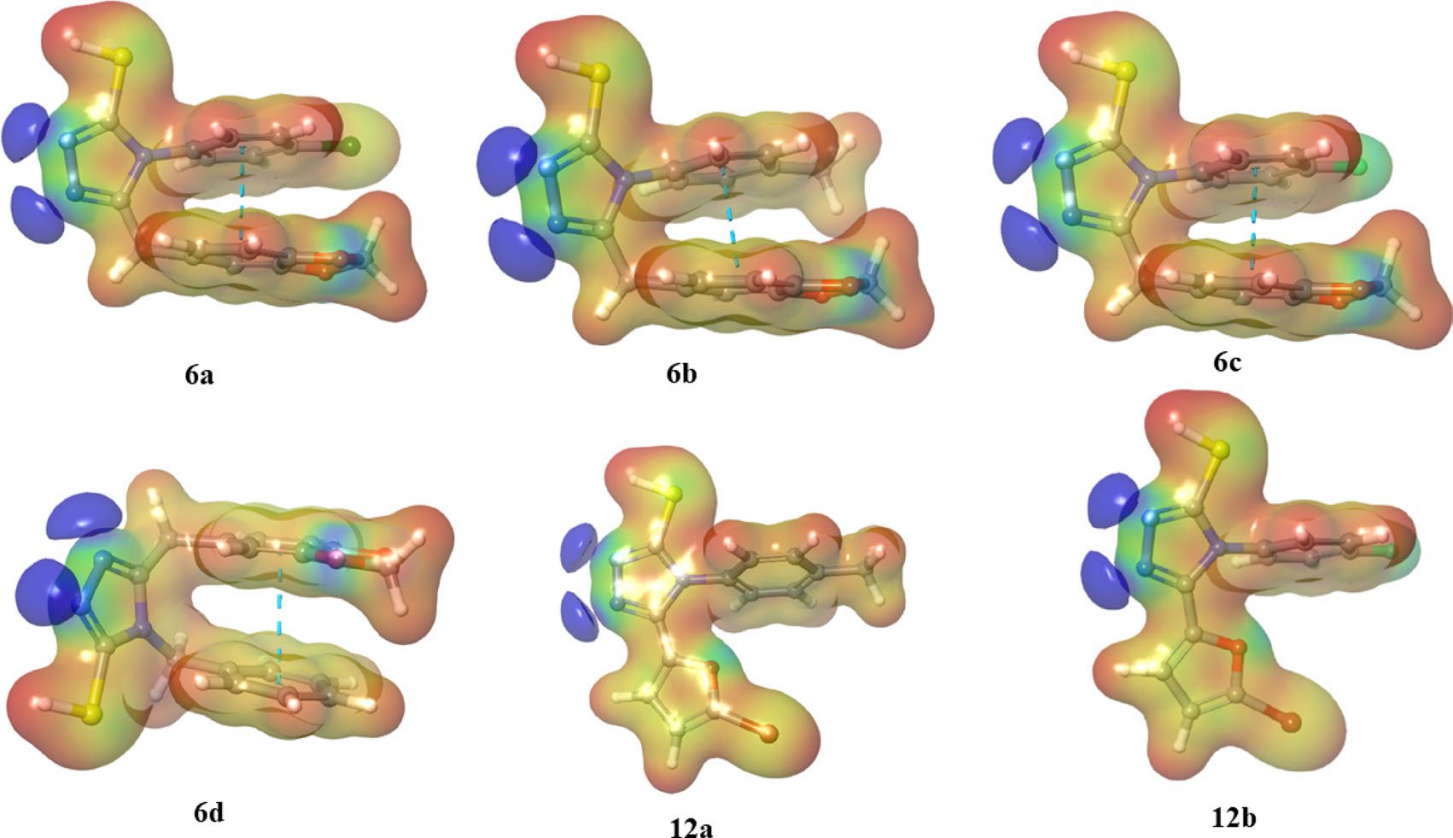
MEPs of triazole derivatives. The image was generated using the Jaguar module of Schrödinger, version 2024-3 (https://www.schrodinger.com/jaguar).

Calculating the molecular electrostatic potential (MEP) in a 3D plot is essential for verifying the inhibitory profiles of compounds. In this study, MEP was carried out using the Jaguar module of Schrödinger 2024 − 3^29^. The MEP, which is based on molecular structure, forecasts physiochemical properties and reveals the size and shape of a molecule’s electrostatic potential. This technique aids in understanding hydrogen bonds and assessing a drug’s susceptibility to electrophilic and nucleophilic attacks^27^. The same methodology and base sets were used to determine the MEPs of the compounds (**6a-d** and **12a-b**). The red areas indicate regions where electrophilic attacks are likely due to a negative charge, whereas the blue areas repel electrophiles. Variations in the electrostatic potential around a compound can affect its binding affinity with the active site. The electrostatic potential mapping around compounds varies on the basis of the types of atoms involved and their electrical properties. This variation may influence binding affinity, making the MEP a useful predictor of a drug’s inhibitory potential. The MEPs of the triazole derivatives (**6a-d** and **12a-b**) are given in Fig. 13.

### Structure‒activity relationships

The anticancer activity of 1,2,4-triazoles against MCF-7 cells tends to occur as follows: **12b** (4-F substituent with a Bromo furan moiety) > **6a** (4-Cl with a Benzo[*d*][1,3] dioxole moiety) > **12a** (4-CH_3_ substituent with a Bromo furan moiety) > **6b** (4-CH_3_ (with a Benzo[*d*][1,3] dioxole moiety) > **6c** (4-F substituent with a Benzo[*d*][1,3] dioxole moiety) > **6d** (benzylic substituent with a Benzo[*d*][1,3] dioxole moiety).

The small size and high electronegativity of fluorine make it the most effective substituent. The maximum activity was observed with the derivative containing a 4-F substituent and a bromo furan moiety, likely due to the hydrophobic interactions of the bromo furan group combined with its strong electronegativity. In contrast, chlorine’s larger size and lower electronegativity may reduce the binding effectiveness, which explains the slightly decreased activity of the benzo[*d*][1,3] dioxole moiety with a 4-Cl substituent. Compared with its 4-Cl counterpart, the 4-F derivative with the benzo[*d*][1,3] dioxole moiety exhibits somewhat reduced activity, possibly due to steric or spatial considerations. On the other hand, the 4-CH_3_ derivatives demonstrated moderate activity. The Bromo furan moiety performs slightly better than the benzo[*d*][1,3] dioxole because of the polarizability and electronegativity associated with the bromine group. The benzylic derivative featuring the benzo[*d*][1,3] dioxole moiety shows the least activity, likely because the bulky group impedes optimal target binding due to steric hindrance. While bulky groups and less electronegative substituents diminish efficacy, the data indicate that electronegative halogens and aromatic systems, particularly bromofuran, play a vital role in enhancing anticancer activity.

A thorough understanding of a compound’s electrical characteristics and potential biological activity requires an awareness of its HOMO–LUMO energy gap (∆E)^27,28^. Among the derivatives studied, the 4-F derivative containing a bromo furan moiety (**12b**) displays the lowest ∆E at 4.6087 eV. Likewise, the 4-Cl derivative featuring a benzo[*d*][1,3]dioxole moiety **(6a)** exhibited an ∆E of 4.62 eV. These values indicate enhanced chemical reactivity as well as superior electron-donating and electron-accepting capabilities, which can be attributed to their good inhibitory effects on the MCF-7 cell line. In contrast, compound **6d**, having the highest ∆E of 5.1421 eV, is the least reactive and most stable among the derivatives.

## Experimental section

###  Materials and experimental procedures

All procured reagents and chemicals were of analytical grade and were obtained from different sources, such as Sigma Aldrich, Spectrochem, Loba Chemie, SRL, and Avra Synthesis, and were used as received without further purification unless otherwise specified; anhydrous conditions were used to carry out the reaction. Melting points were measured with open capillary tubes and reported uncorrected. The progression of the reaction was tracked via aluminum sheets (precoated) with a mobile phase of toluene and ethyl acetate (3:1), with visualization in a UV cabinet. A Shimadzu ATR Spirit analyser was used to obtain infrared radiation data in the 4000–400 cm^−1^ range. Both ^1^H and ^13^C NMR spectra were recorded on a Bruker AM 400 MHz spectrometer. TMS was employed as an internal standard, DMSO-*d*_6_ was used as a solvent for NMR analysis, and a Waters ARC with a 2998 SQ detector 2 was used to collect the ESI + m/z values of selected fragments (information provided in the supplementary material). The MCF-7 breast cancer cell line was obtained from the National Centre for Cell Science (NCCS), Pune, India, while fetal bovine serum (FBS) and other reagents were sourced from HiMedia, Mumbai.

### Chemistry

#### **Procedure for the synthesis of esters of 2-(benzo[***d*]dioxol-5-yl)acetic acid and 5-bromofuran-2-carboxylic acid (2 and 8)

2-(benzo[*d*]dioxol-5-yl)acetic acid and 5-bromofuran-2-carboxylic acid (**1** and **7**, 0.05 mol) were dissolved in absolute ethanol, after which a catalytic amount of sulfuric acid was added. The mixture was then refluxed for six to eight hours. Thin-layer chromatography was employed to monitor the progress of the reaction. Upon completion and cooling of the reaction, the mixture was added to crushed ice and washed with a bicarbonate solution. The product was subsequently extracted with ethyl acetate, and the solvent was evaporated under reduced pressure.

#### Procedure for the synthesis of 2-(benzo[*d*]dioxol-5-yl)acetohydrazide and 5-bromofuran-2-carbohydrazide (3 and 9)

A solution of esters derived from 2-(benzo[*d*]dioxol-5-yl)acetic acid and 5-bromofuran-2-carboxylic acid (1 mmol) was prepared in absolute ethanol, utilizing an equimolar amount of hydrazine hydrate. The reaction mixture was refluxed for approximately four to five hours, and the progress was monitored by thin-layer chromatography using a mobile phase composed of toluene and ethyl acetate in a 3:1 ratio. Upon completion of the reaction, excess hydrazine hydrate was removed under vacuum. The mixture was then cooled to room temperature, which induced solidification of the product. The mixture was subsequently filtered, rinsed with ice-cold water, and recrystallized with ethyl acetate.

#### Procedure for the synthesis of 2-(2-(benzo[*d*][1,3]dioxol)acetyl)-*N*-substituted phenylhydrazine-1-carbothioamides and (5-bromofuran-2-carbonyl)-***N***-substituted phenyl hydrazine-1-carbothiamides (5a-d and 11a-b)

Equimolar solutions of 2-(benzo[*d*]dioxol-5-yl)acetohydrazide and 5-bromofuran-2-carbohydrazide in ethanol were refluxed with various phenyl isothiocyanates for approximately six to seven hours. The reaction progress was monitored using thin-layer chromatography. The obtained product was hot-filtered and used for the next step without further purification.

####  Procedure for the synthesis of 5-(benzo[*d*][1,3]dioxol-5-ylmethyl)-4***H-***1,2,4-triazole-3-thiols and 5-(5-bromofuran-2-yl)-4***H***-1,**2**,4-triazole-3-thiols (6a-d and 12a-b)

Intermediates **5a-d** and **11a-b** were treated with an equimolar solution of 4 N sodium hydroxide and refluxed in ethanol for three to four hours, with the reaction progress monitored by TLC. Once the reaction was complete, the mixtures were neutralized with 4 N hydrochloric acid, and the pH was adjusted to 2–3 to facilitate product isolation. The resulting product was then filtered and recrystallized from ethanol.

### Spectral analysis

####  5-(benzo[*d*][1,3]dioxol-5-ylmethyl)-4-(4-chlorophenyl)-4*H*-1,2,4-triazole-3-thiol (6a)

Buff solid. Yield: 86%. M.P: > 200 °C. FT-IR (ν cm^− 1^): 778 (C–Cl), 2762 (SH), 1634 (C=N), 1494 (C=C), 1090 (C–O), 2927 (aliphatic C–H), 3034 (aromatic C–H). ^1^H NMR (400 MHz, DMSO-*d*_6,_ ppm): δ 3.7 (s, 2 H), δ 5.9 (s, 2 H), δ 6.39 (d, H, *J* = 8 Hz), δ 6.5 (s, H), δ 6.73 (d. H, *J* = 8.0 Hz), δ 7.28 (d, 2 H, *J* = 8.4 Hz), δ 7.57 (d, 2 H, *J* = 8.4 Hz), δ 13.82 (s, H). ^13^C NMR (100 MHz, DMSO-*d*_6,_ ppm): δ 31.47, 101.35, 108.52, 109.57, 122.33, 128.36, 129.78, 130.74, 132.95, 134.56, 146.54, 147.68, 151.80, 168.36. Molecular formula: [C_16_H_12_ClN_3_O_2_S]. ESI mass (m/z): 346.19 (M + H)^+^.

####  5-(benzo[*d*][1,3]dioxol-5-ylmethyl)-4-(*P*-tolyl)-4*H*-1,2,4-triazole-3-thiol (6b)

Buff solid. Yield: 80%. M.P: >200 °C. FT-IR (ν cm^− 1^): 1080 (C–O), 1490 (C=C), 1641 (C=N), 2756 (SH), 2903 (aliphatic C–H), 3031 (aromatic C–H). ^1^H NMR (400 MHz, DMSO-*d*_6,_ ppm): δ 2.5 (s, 3 H), δ 3.7 (s, 2 H), δ 5.9 (s, 2 H), δ 6.39 (d, H, *J* = 7.2 Hz), δ 6.52 (s, H), δ 6.73 (d. H, *J* = 7.6 Hz), δ 7.12 (d, 2 H, *J* = 6.4 Hz), δ 7.30 (d, 2 H, *J* = 6.8 Hz), δ 13.75 (s, H). ^13^C NMR (100 MHz, DMSO-*d*_6,_ ppm): δ 21.24, 31.46, 101.34, 108.50, 109.57, 122.27, 128.49, 128.59, 130.20, 131.46, 139.51, 146.51, 147.64, 151.96, 168.38. Molecular formula: [C_17_H_15_N_3_O_2_S]. ESI mass (m/z): 326.23 (M + H)^+^.

#### 5-(benzo[*d*][1,3]dioxol-5-ylmethyl)-4-(4-fluorophenyl)-4*H*-1,2,4-triazole-3-thiol (6c)

White solid. Yield: 83%. M.P: >200 °C. FT-IR (ν cm^− 1^): 1247 (C–F), 2757 (SH), 1600 (C=N), 1485 (C=C), 1072 (C–O), 2899 (aliphatic C–H), 3030 (aromatic C–H). ^1^H NMR (400 MHz, DMSO-*d*_6,_ ppm): δ 3.7 (s, 2 H), δ 5.9 (s, 2 H), δ 6.37 (d, H, *J* = 8.0 Hz), δ 6.51 (d, H, *J* = 1.6 Hz), δ 6.73 (d. H, *J* = 8.0 Hz), δ 7.28–7.36 (m, 4 H), δ 13.8 (s, H). ^13^C NMR (100 MHz, DMSO-*d*_6,_ ppm): δ 31.50, 101.35, 108.52, 109.52, 116.54, 122.30, 128.39, 130.35, 131.20, 146.54, 147.67, 151.94, 161.37, 163.82, 168.51. Molecular formula: [C_16_H_12_FN_3_O_2_S]. ESI mass (m/z): 330.22 (M + H)^+^.

####  5-(benzo[*d*][1,3]dioxol-5-ylmethyl)-4-benzyl-4*H*-1,2,4-triazole-3-thiol (6d)

White solid. Yield: 89%. M.P: 140–143 °C. FT-IR (ν cm^− 1^): 2763 (SH), 1617 (C=N), 1494 (C=C), 1093 (C–O), 2929 (aliphatic C–H), 3034 (aromatic C–H). ^1^H NMR (400 MHz, DMSO-*d*_6,_ ppm): δ 3.8 (s, 2 H), δ 5.1 (s, 2 H), δ 5.9 (s, 2 H), δ 6.53 (d, H, *J* = 8.0 Hz), δ 6.61 (d. H, *J* = 1.2 Hz), δ 6.73 (d, H, *J* = 7.6 Hz), δ 7.14 (d, 2 H, *J* = 6.4 Hz), δ 7.25–7.31 (m, 3 H), δ 13.77 (s, H). ^13^C NMR (100 MHz, DMSO-*d*_6,_ ppm): δ 31.13, 46.20, 101.37, 108.62, 109.62, 122.34, 127.27, 127.98, 128.34, 128.97, 135.91, 146.63, 147.74, 152.06, 167.96. Molecular formula: [C_17_H_15_N_3_O_2_S]. ESI mass (m/z): 326.24 (M + H)^+^.

#### 5-(5-bromofuran-2-yl)-4-(*p*-tolyl)-4*H*-1,2,4-triazole-3-thiol (12a)

White solid. Yield: 75%. M.P: 190–193 °C. FT-IR (ν cm^− 1^): 561 (C-Br), 2752 (SH), 1675 (C=N), 1494 (C=C), 1086 (C–O), 2930 (aliphatic C–H), 3104 (aromatic C–H). ^1^H NMR (400 MHz, DMSO-*d*_6,_ ppm): δ 2.11 (s, 3 H), δ 6.80 (d, H, *J* = 3.6 Hz), δ 7.25 (d, H, *J* = 3.6 Hz), δ 7.48 (d, 2 H, *J* = 8.8 Hz), δ 7.63 (d. 2 H, *J* = 8.8 Hz), δ 13.70 (s, H). ^13^C NMR (100 MHz, DMSO-*d*_6,_ ppm): δ 12.61, 114.81, 120.44, 129.91, 130.64, 131.18, 133.18, 134.55, 149.59, 158.73. Molecular formula: [C_13_H_10_BrN_3_OS]. ESI mass (m/z): 358.07 (M + Na)^+^.

#### 5-(5-bromofuran-2-yl)-4-(4-fluorophenyl)-4*H*-1,2,4-triazole-3-thiol (12b)

White solid. Yield: 83%. M.P: >200 °C. FT-IR (ν cm^− 1^): 466 (C-Br), 1068 (C–O), 1220 (C–F), 1455 (C=C), 1621 (C=N), 2971 (aliphatic C–H), 3075 (aromatic C–H). ^1^H NMR (400 MHz, DMSO-*d*_6,_ ppm): δ 5.98 (d, H, *J* = 3.6 Hz), δ 6.64 (d, H, *J* = 3.6 Hz), δ 7.44 (t, 2 H, *J* = 8.4 Hz), δ 7.52–7.56 (m. 2 H), δ 14.24 (s, H). ^13^C NMR (100 MHz, DMSO-*d*_6,_ ppm): δ 114.34, 115.44, 116.97, 117.20, 125.34, 130.78, 131.56, 142.70, 161.82, 169.10. Molecular formula: [C_12_H_7_BrFN_3_OS]. ESI mass (m/z): 340.11 (M + H)^+^.

### Biological evaluation

#### Cell culture

The MCF-7 cell lines were sourced from the National Centre for Cell Science (NCCS, Pune, India). These cell lines were maintained in Minimal Essential Media (MEM) supplemented with 10% Fetal bovine serum (FBS) and 1% antibiotic solution, maintained under standard culture conditions at 37 °C in a humidified atmosphere containing 5% CO_2_.

#### MTT assay

The cells (5,000/well) were seeded in 96-well plates (flat bottom) and incubated at 37 °C with 5% CO₂ overnight. Culture medium (without serum) was used for further treatment. Stock solutions of the synthesized compounds were prepared in DMSO and diluted to various concentrations using culture medium. Cells were then treated with these dilutions and incubated for 48 h. Following treatment, 20 µL of MTT solution (5 mg/mL in PBS) was added to each well, and the mixture was incubated for 4 h. The culture medium was slowly removed, the resulting formazan crystals were solubilized in 200 µL of DMSO, and the absorbance was read at 570 nm to determine % inhibition^27^. The formula applied to calculate the % inhibition is as follows:

% Inhibition = (1- OD of Test/OD of Control) × 100.

###  Computational studies

#### Molecular docking

A molecular docking study was performed utilizing the Glide module of Schrödinger 2024-3^21^. This involved designing compounds *via* a 2D sketcher and preparing them in LigPrep. The required protein was sourced from the Protein Data Bank using its PDB ID and underwent preparation. Ultimately, the prepared ligands and protein were docked together. The docking scores and interactions of the compounds with the protein’s amino acid residues were carefully considered for the analysis.

#### Molecular dynamics

The Desmond module of the Schrödinger 2024-3 was employed to conduct molecular dynamics simulations to further stabilize the docked complex **12b**^25^. The SPC solvent model was constructed using the system builder tool by enclosing the docked complex within an orthorhombic box. After minimization, salts were added to the model. The system was then loaded into the molecular dynamics tool in the Desmond module from the workspace. A simulation was conducted for 200 ns utilizing the NPT ensemble at a temperature of 300 K and a pressure of 1.01325 bar, employing the OPLS4 force field. The obtained simulated trajectories were analysed using a simulation interaction diagram, and the results were interpreted.

#### DFT studies

The structure of the molecules was optimized using the hybrid functional B3LYP-D_3_ for theory and the 6-31Gss basis set of the Jaguar module of the Schrodinger materials suite^29^. The harmonic frequency calculation followed the optimization to ensure that the structure was at the global minimum of the potential energy surface. The optimized structure was utilized for the calculation of the HOMO and LUMO.

## Conclusion

We successfully synthesized and characterized a new series of benzo[*d*][1,3]dioxole and 5-bromofuran-tethered 1,2,4-triazole hybrids, achieving good yields. These derivatives were evaluated for their in vitro anticancer potential against the MCF-7 cell line. Compound **12b** demonstrated an IC_50_ value of 3.54 ± 0.265 µg/mL against MCF-7 cells. Additionally, we performed in silico molecular docking to predict the orientation of the compounds, conducted molecular dynamics simulations to assess the binding stability with the target protein, and utilized DFT to determine the energy gap of the FMOs and the MEP to identify sites for nucleophilic and electrophilic attacks. The experimental data and structure‒activity relationship (SAR) studies indicate substantial activity in the derivatives featuring halogen substituents. The findings of this study underscore the potential of benzo[*d*][1,3]dioxole and 5-bromofuran, particularly when combined with 1,2,4-triazole, to create hybrid compounds that could serve as promising candidates for future cancer research targeting the MCF-7 breast cancer cell line. These results indicate that these compounds may significantly contribute to advancing targeted cancer therapies and pave the way for new avenues of investigation within the field of oncology.

## Electronic supplementary material

Below is the link to the electronic supplementary material.

Supplementary Material 1.

## Data Availability

Data is provided within the manuscript or supplementary information files.
