# Supplementary material for: Design, synthesis and evaluation of benzodioxole and bromofuran tethered 1,2,4-triazole hybrids as potential anti breast cancer agents with computational insights
Source: Sci Rep. 2025 Jul 16;15:25680. doi: 10.1038/s41598-025-09420-1 (PMC12263850; doi:10.1038/s41598-025-09420-1)
Supplement: Supplementary file 1 — Supplementary Material 1. [file 41598_2025_9420_MOESM1_ESM.docx]

**Supplementary data**

 **
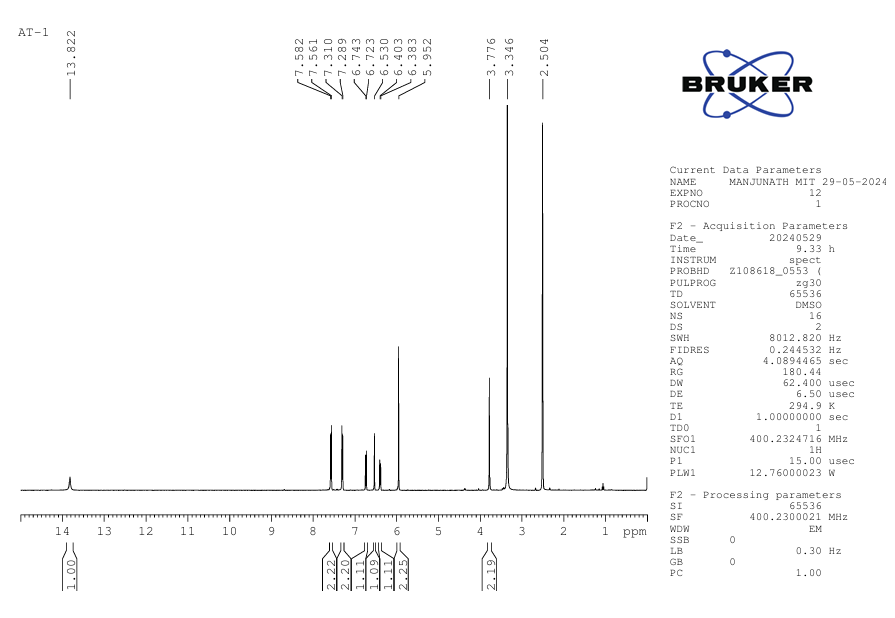
**

**Fig. S1** ^1^H NMR of **6a**


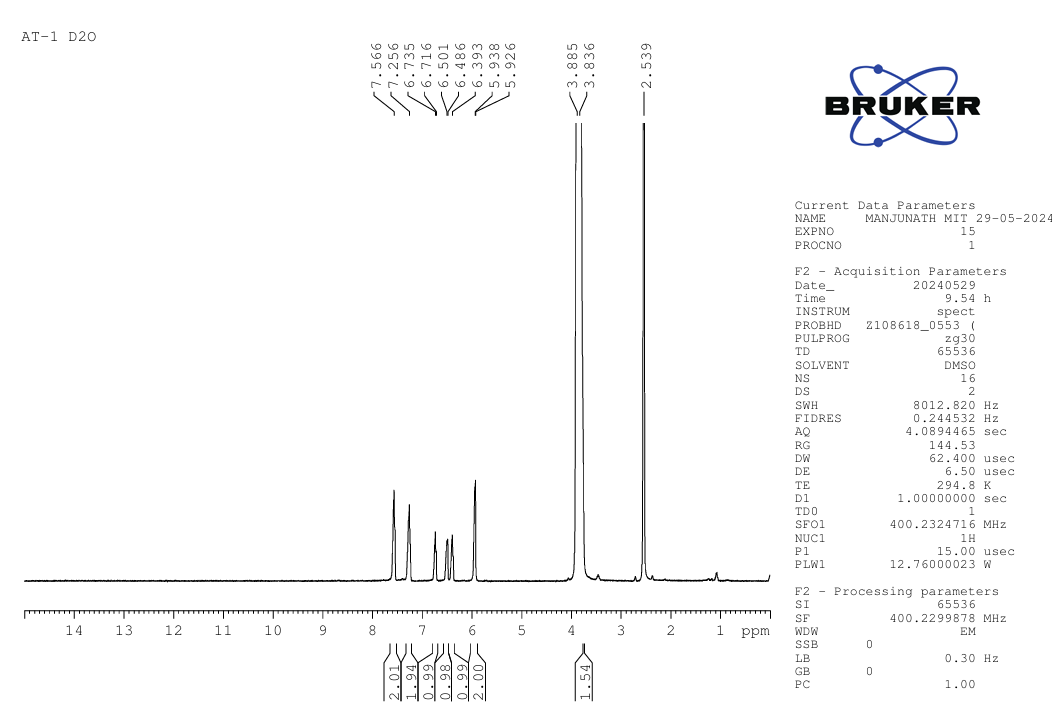


**Fig. S2** ^1^H NMR of **6a** with D_2_O exchange

**
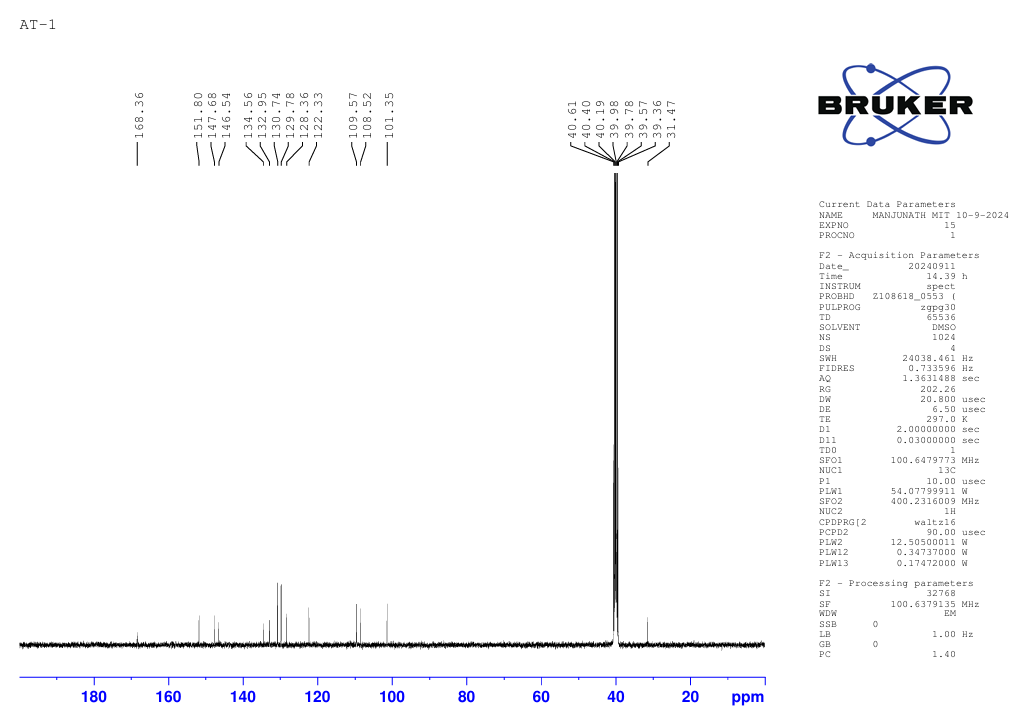
**

**Fig. S3** ^13^C NMR of **6a**

**
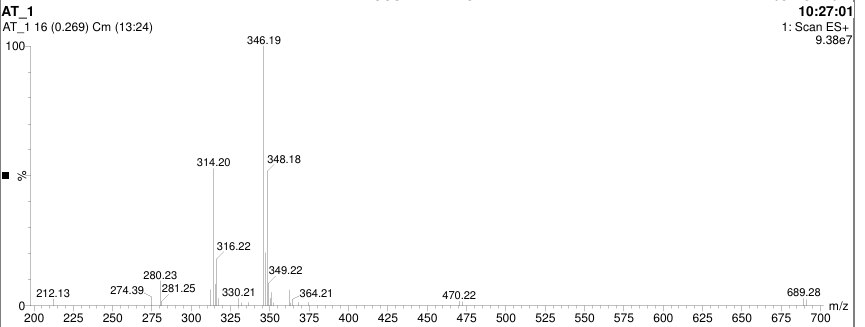
**

**Fig. S4** Mass of **6a**

**
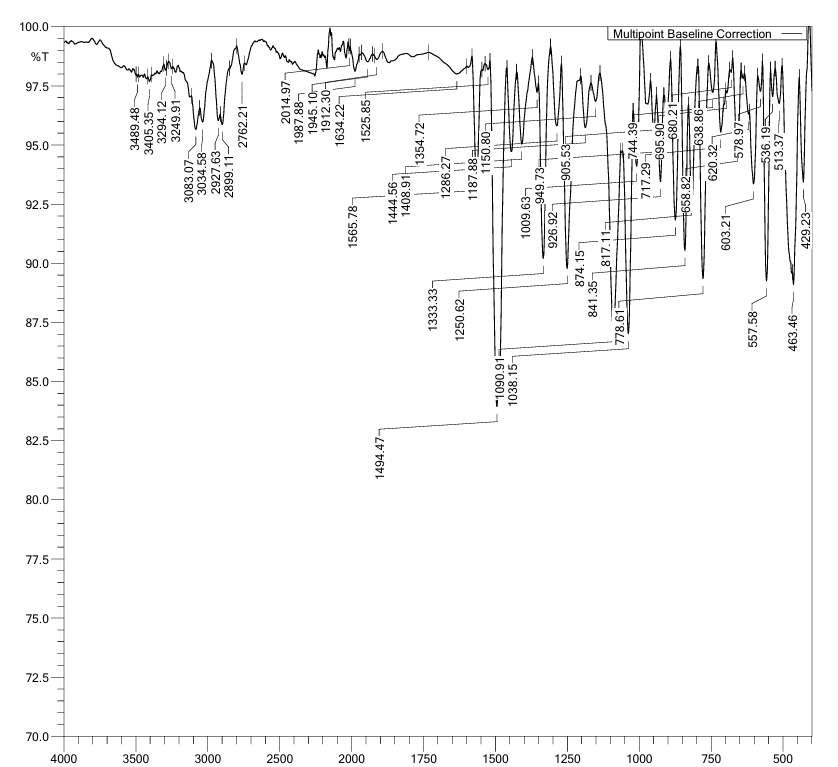
**

**Fig. S5**  FTIR spectra of **6a**

 **
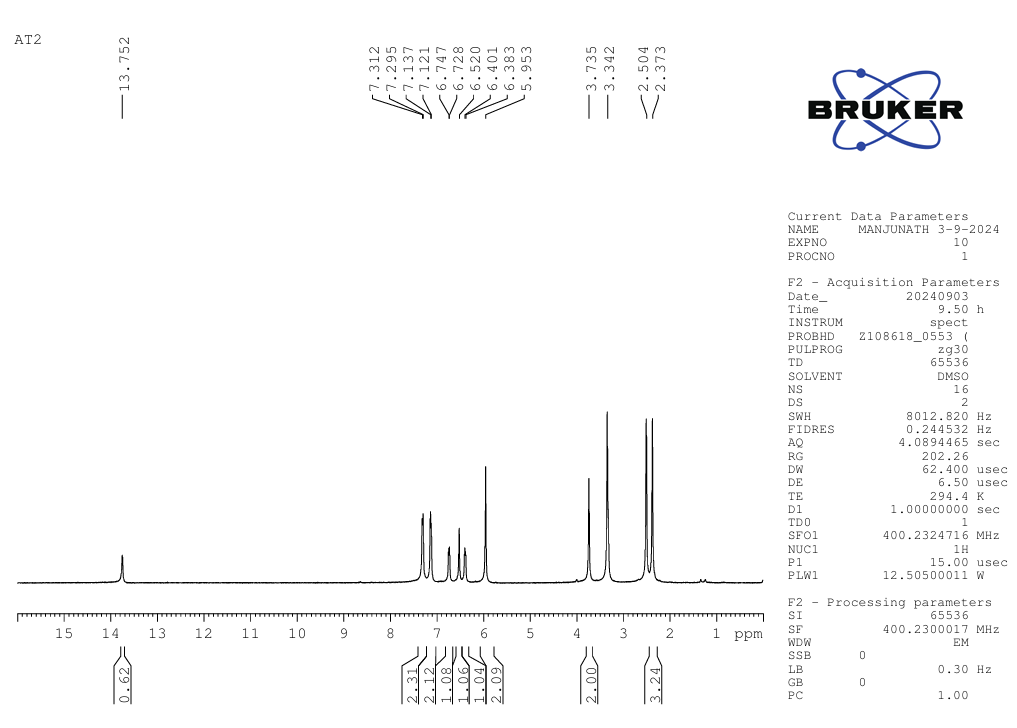
**

**Fig. S6** ^1^H NMR of **6b**


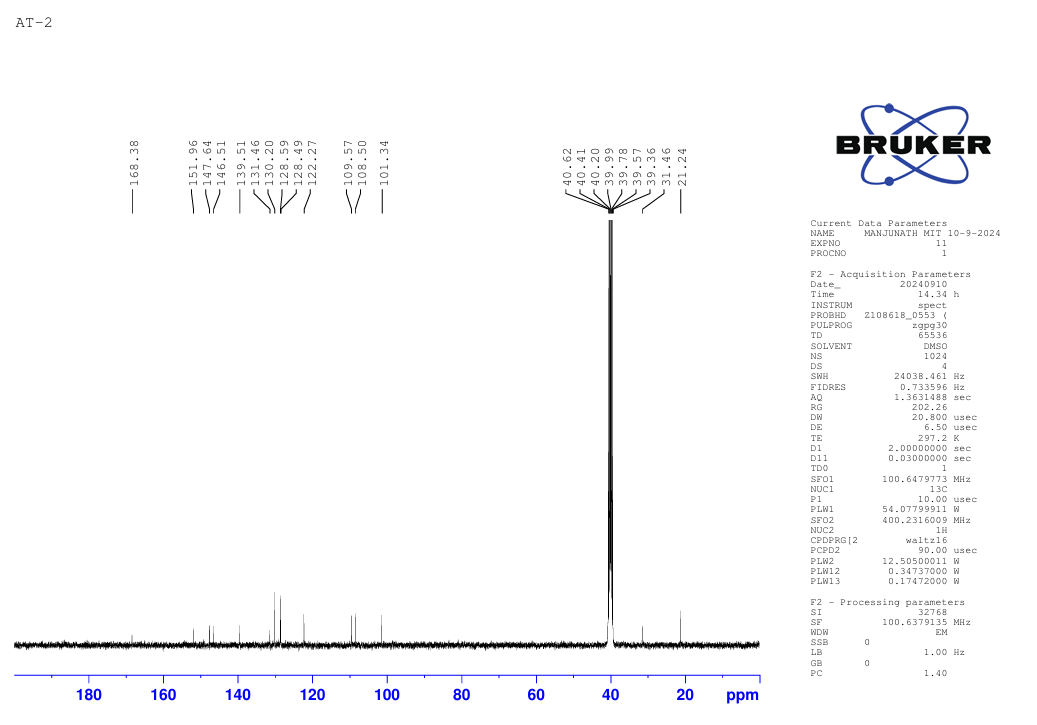


**Fig. S7** ^13^C NMR of **6b**


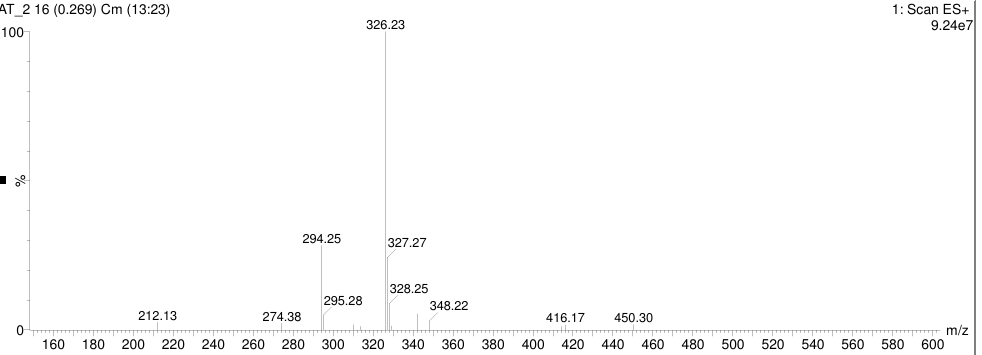


**Fig. S8** Mass of **6b**

**
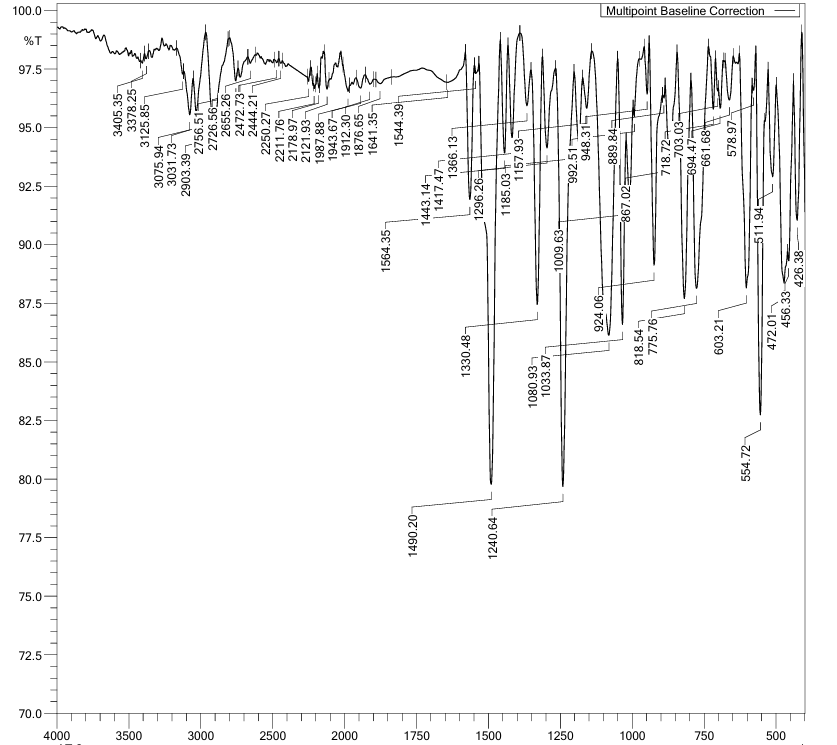
**

**Fig. S9** FTIR of **6b**


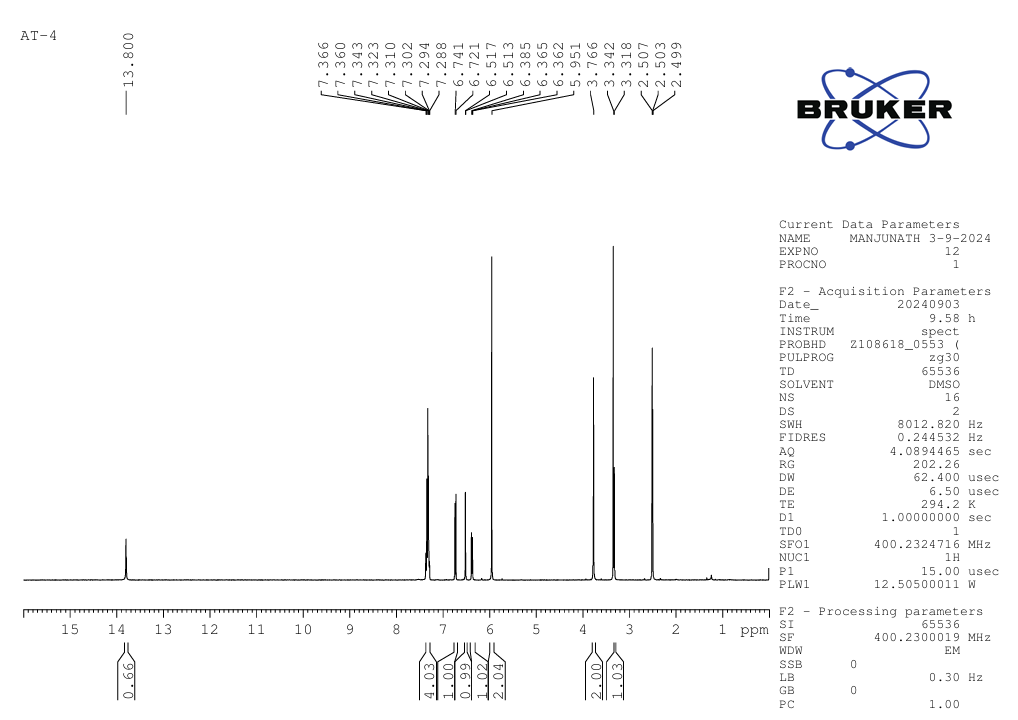


**Fig. S10** ^1^H NMR of **6c**

 **
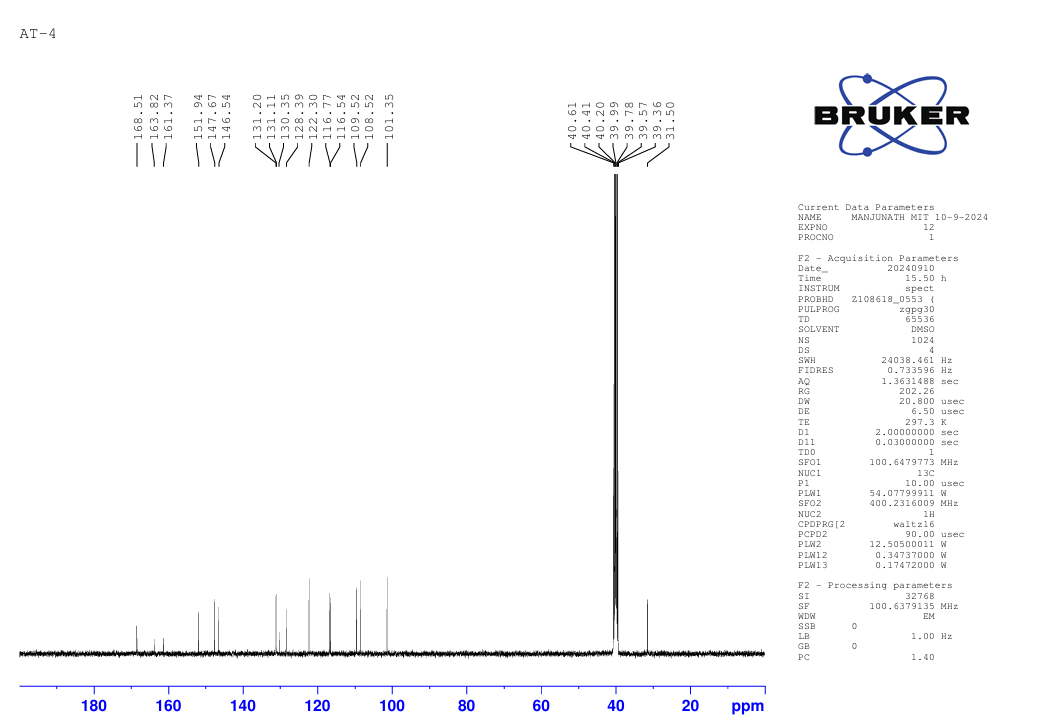
**

**Fig. S11** ^13^C NMR of **6c**


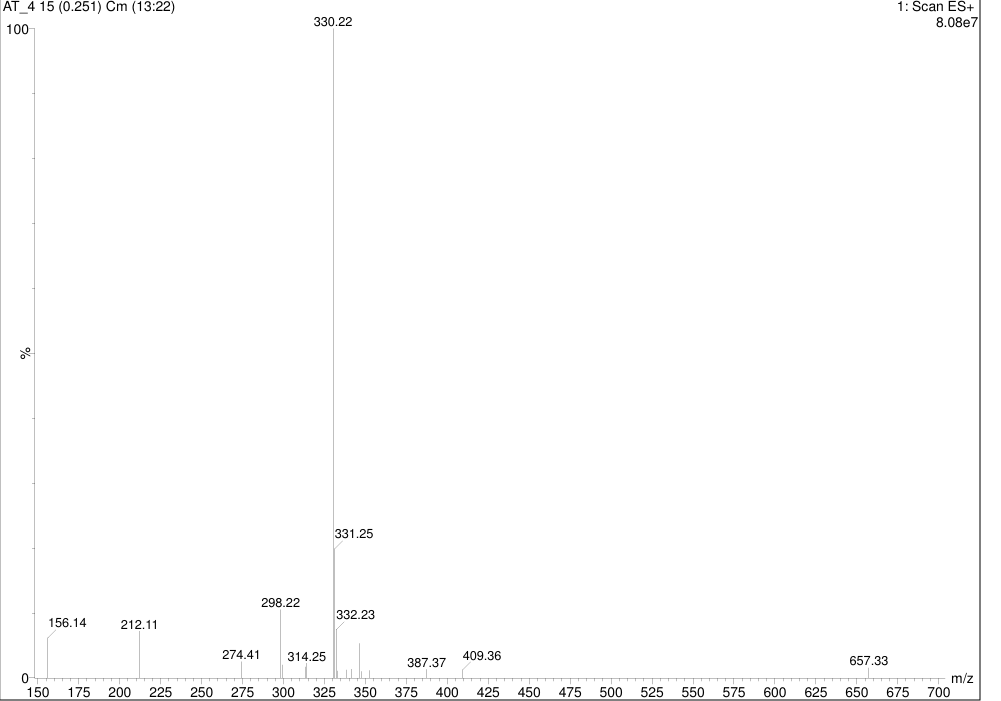


**Fig. S12** Mass of **6c**

**
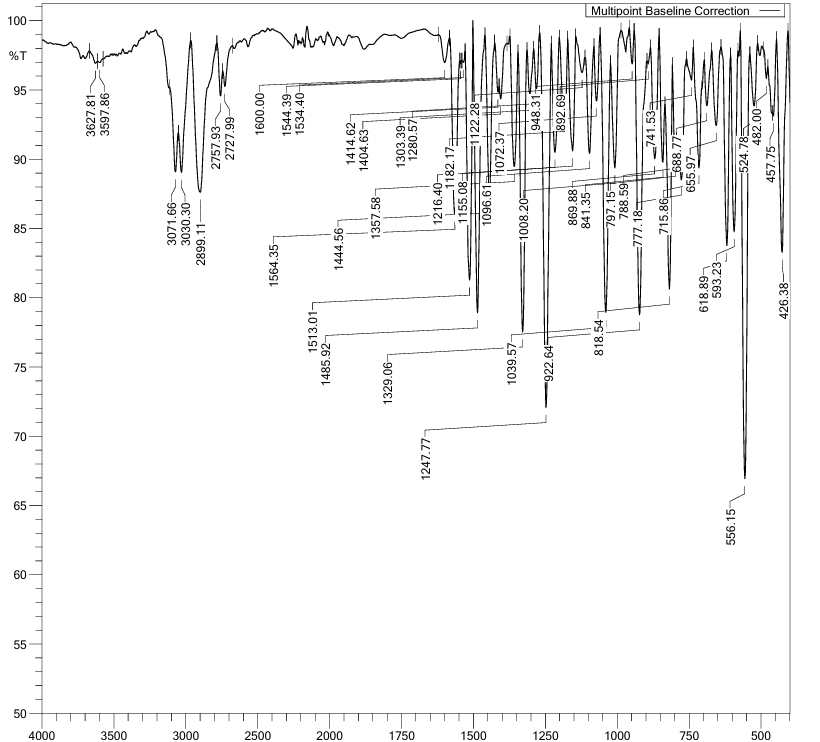
**

**Fig. S13** FTIR of **6c**


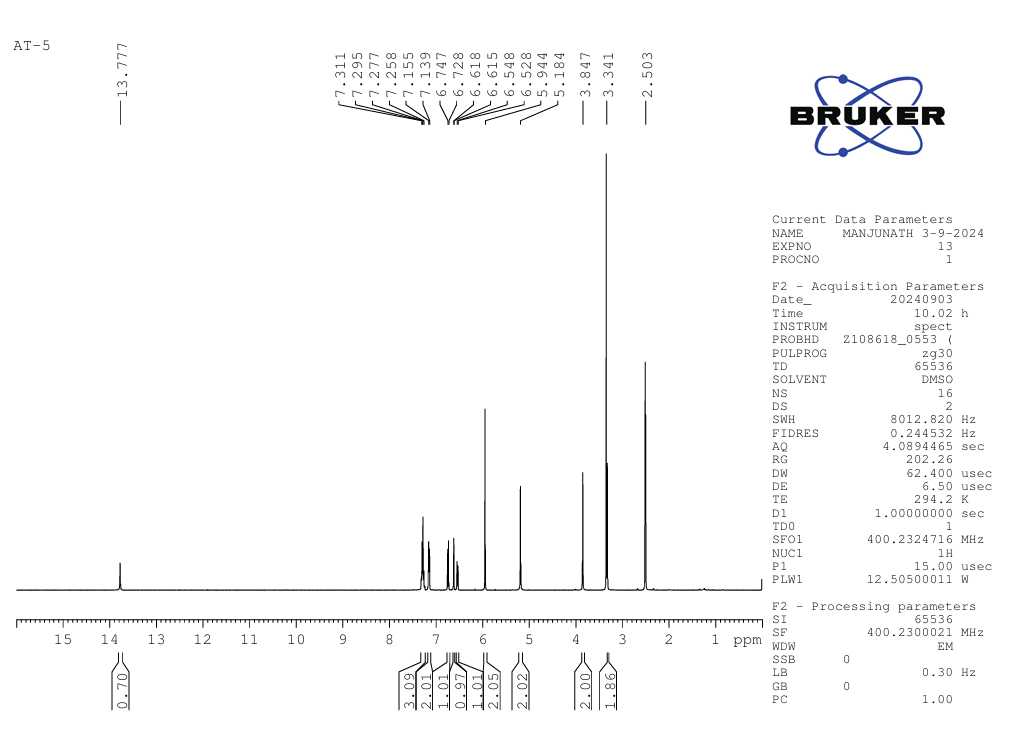


**Fig. S14** ^1^H NMR of **6d**


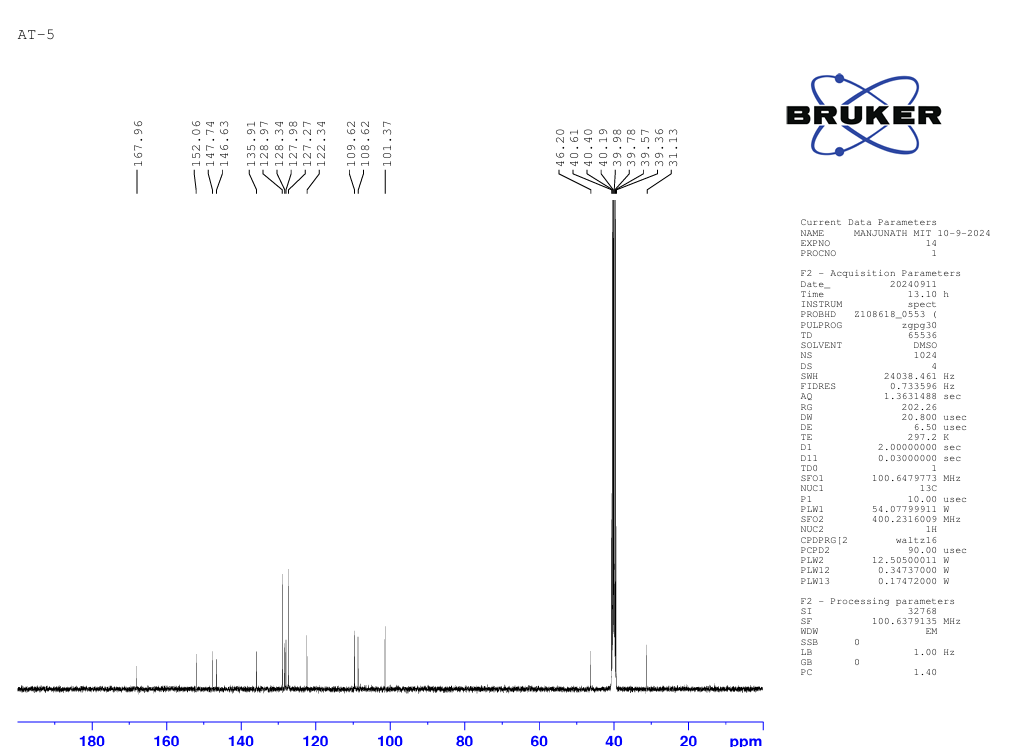


**Fig. S15** ^13^C NMR of **6d**

**
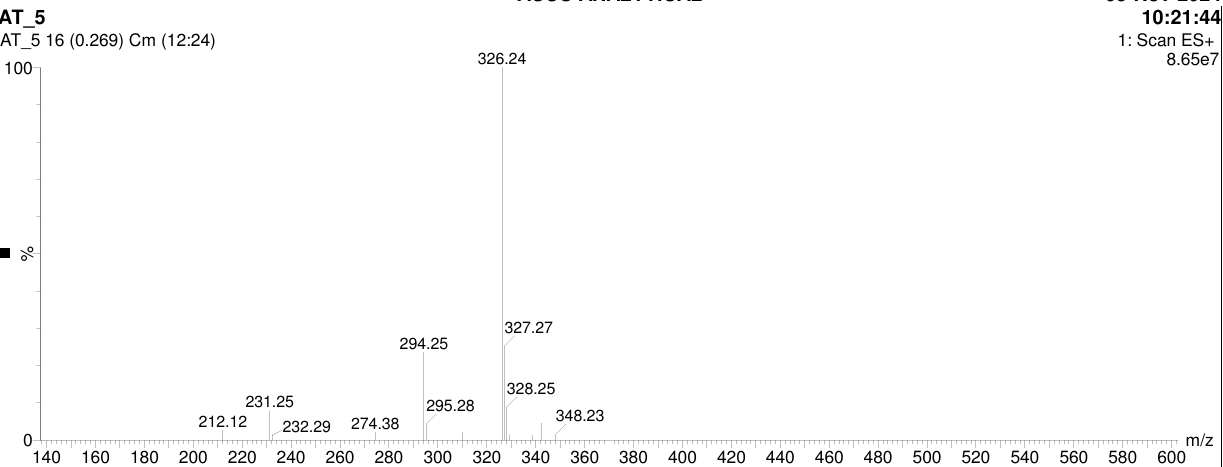
**

**Fig. S16** Mass of **6d**

**
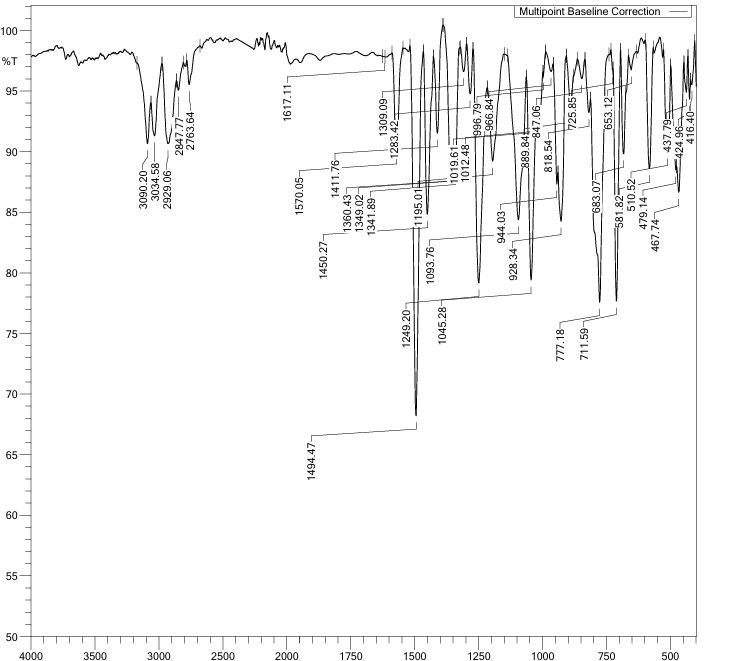
**

**Fig. S17** FTIR of **6d**


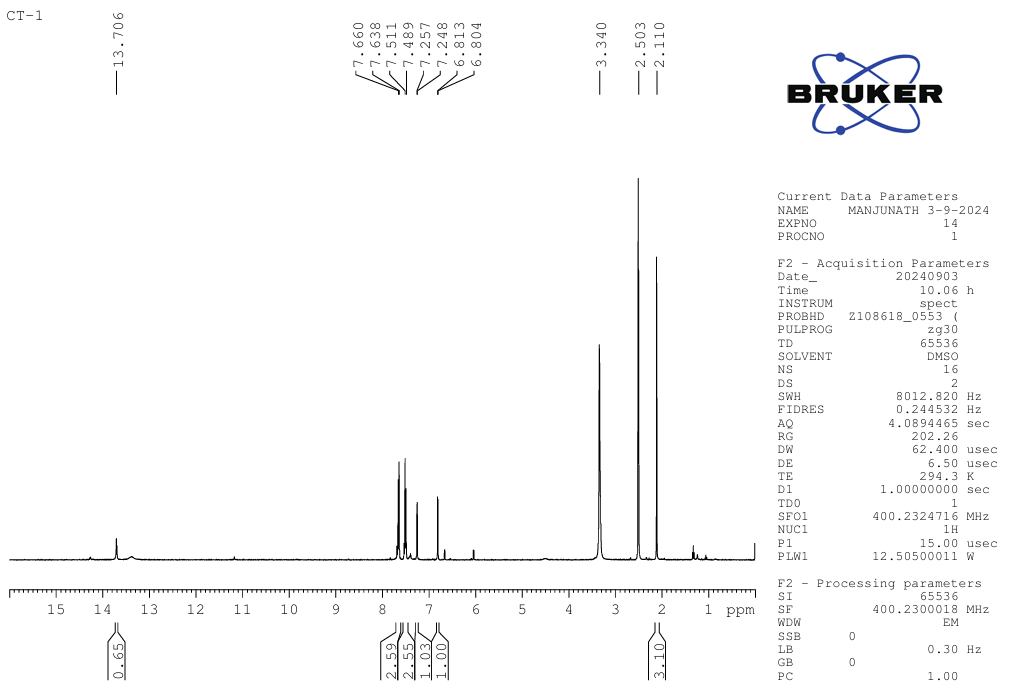


**Fig. S18** ^1^H NMR of **12a**


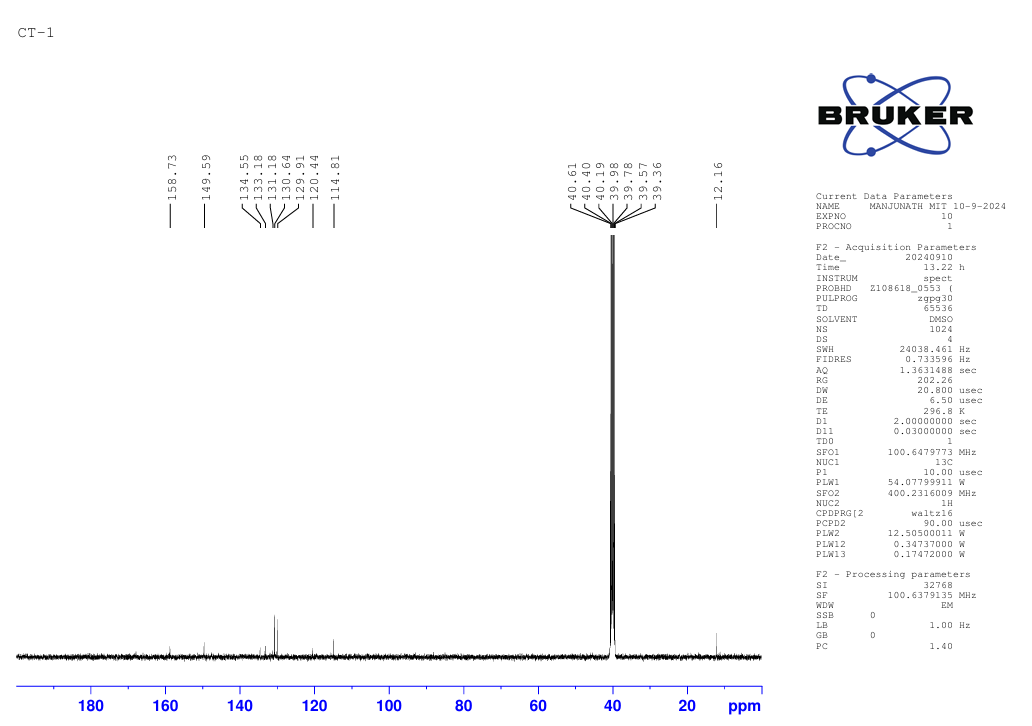


**Fig. S19** ^13^C NMR of **12a**

**
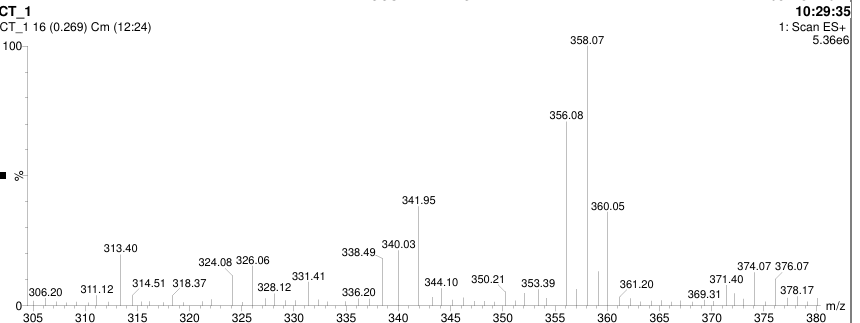
**

**Fig. S20** Mass of **12a**

**
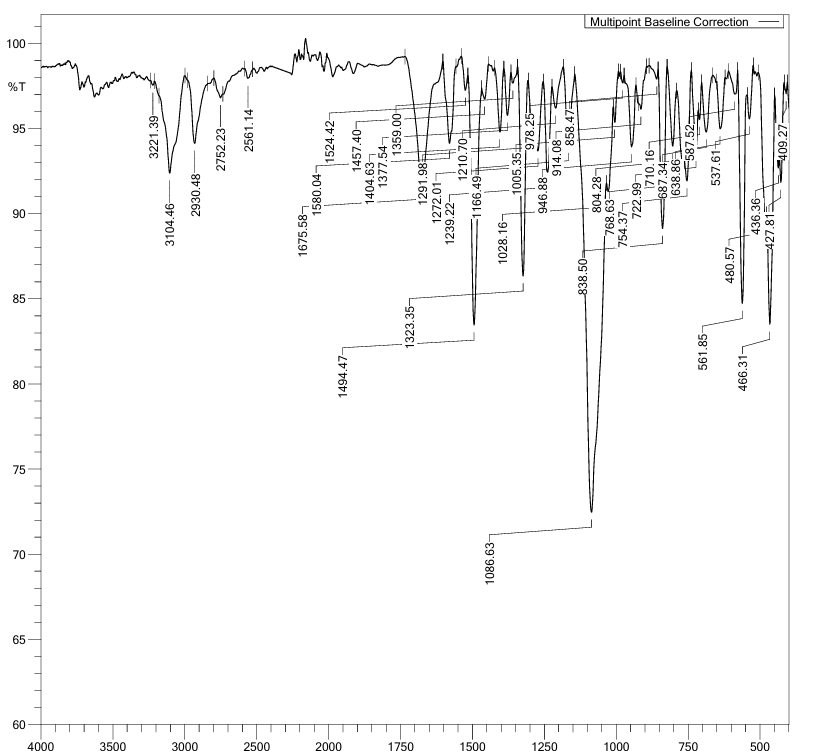
**

**Fig. S21** FTIR of **12a**


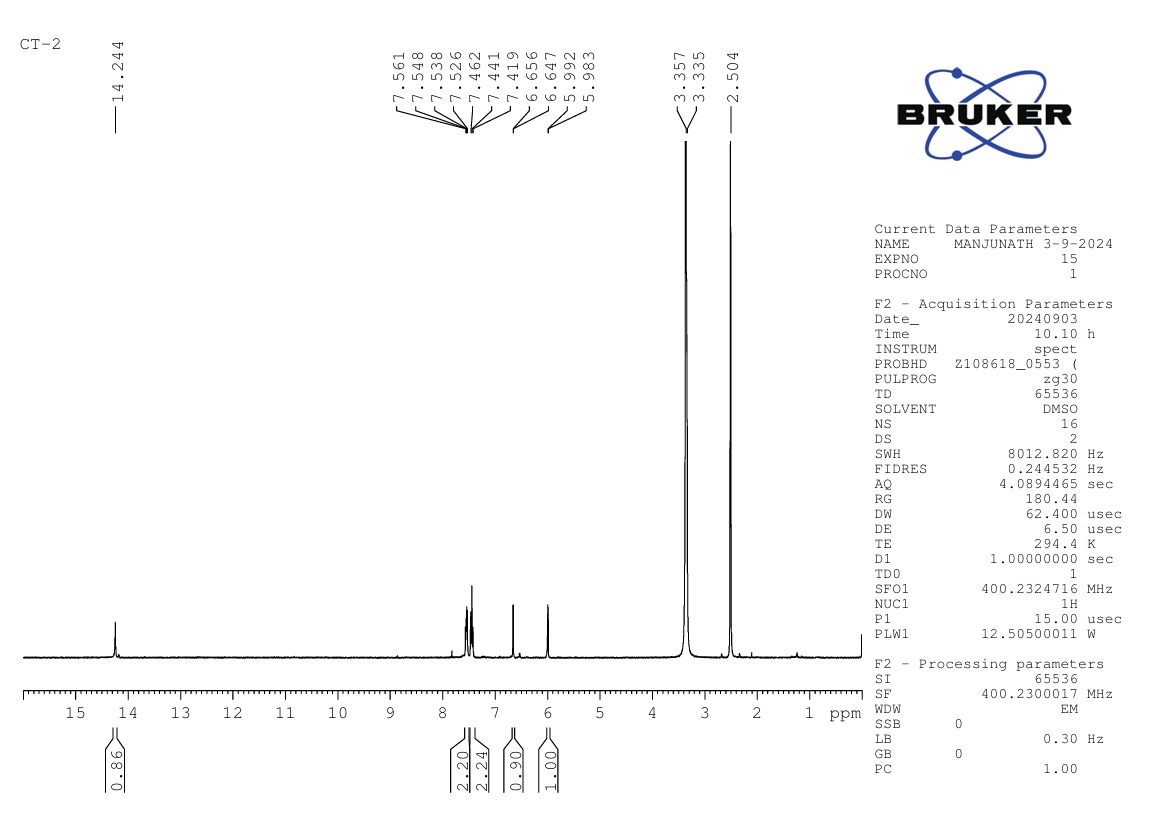


**Fig. S22** ^1^H NMR of **12b**


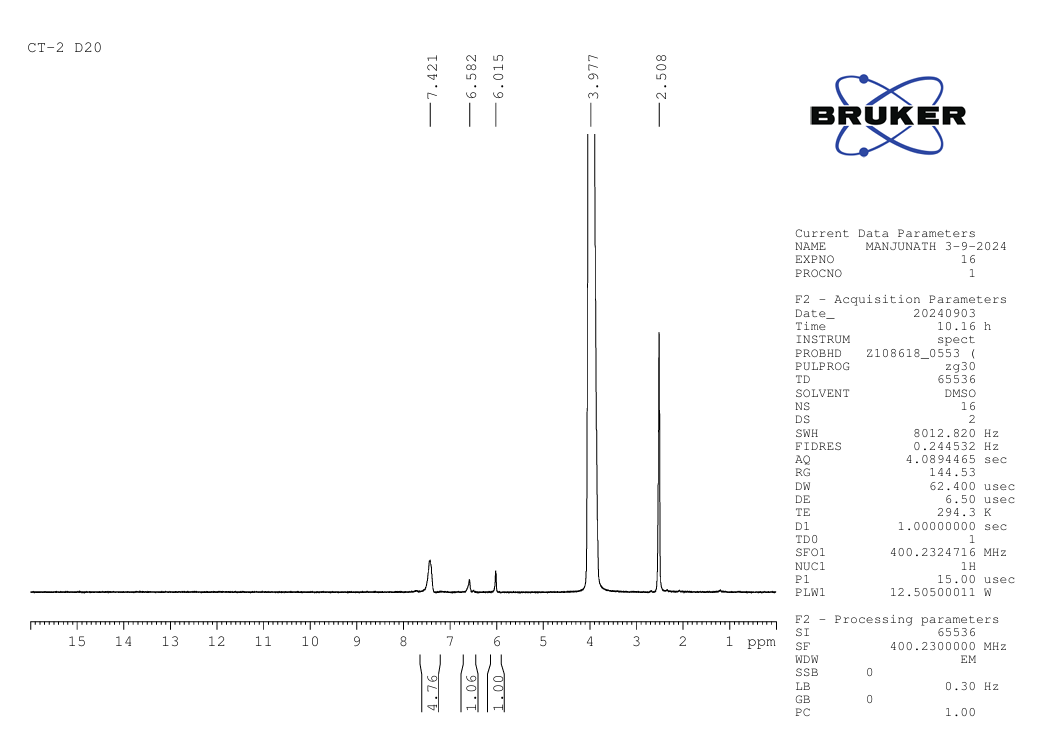


**Fig. S23** ^1^H NMR of **12b** with D_2_O exchange


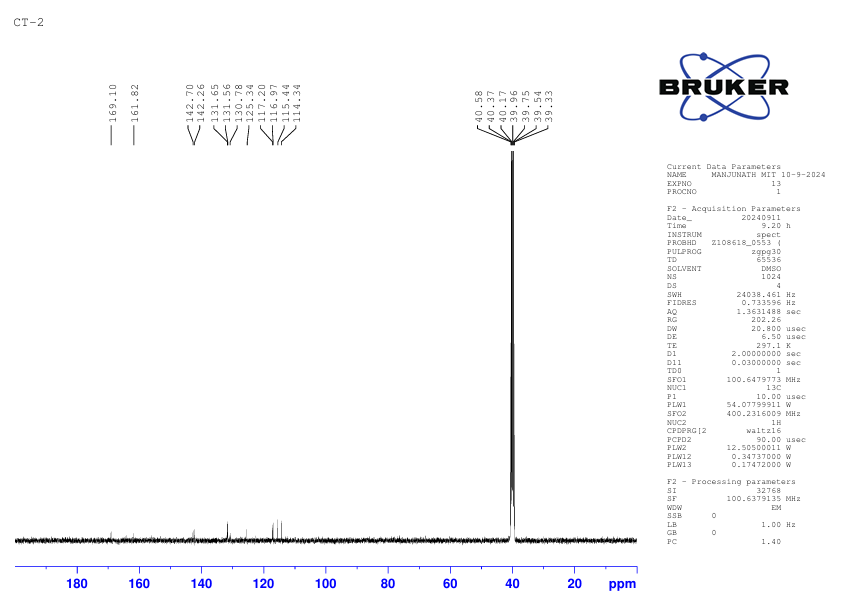


**Fig. S24** ^13^C NMR of **12b**


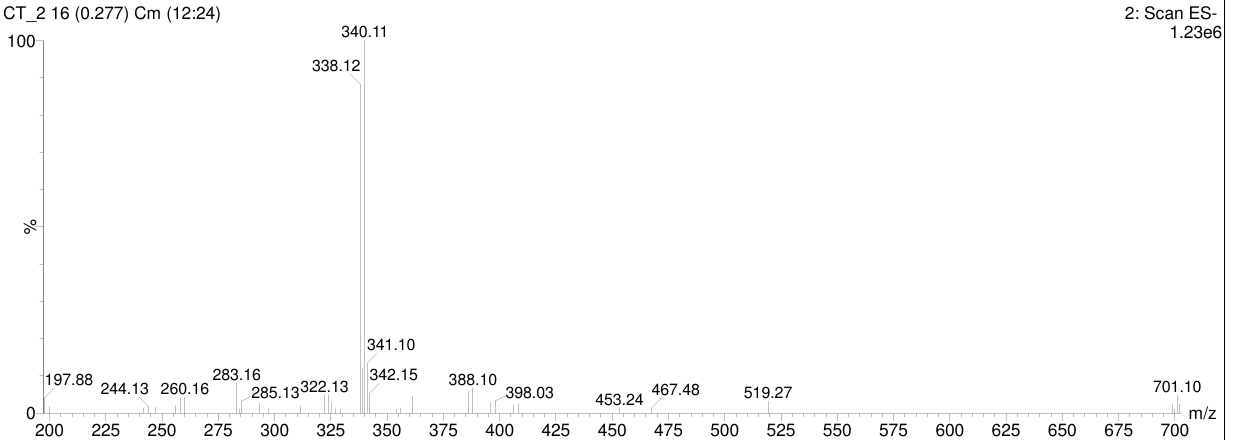


**Fig. S25** Mass of **12b**


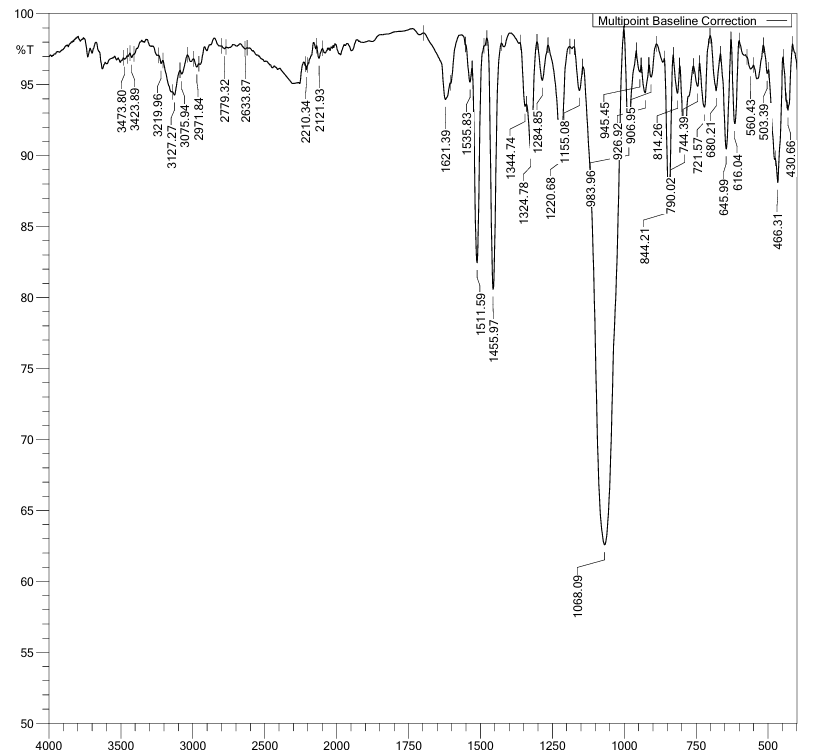


**Fig. S26** FTIR of **12b**

**2.3 Computational studies**

**2.3.1 Molecular docking**


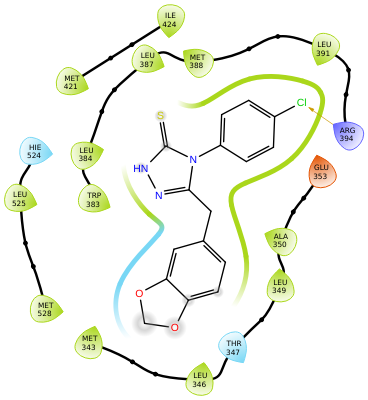

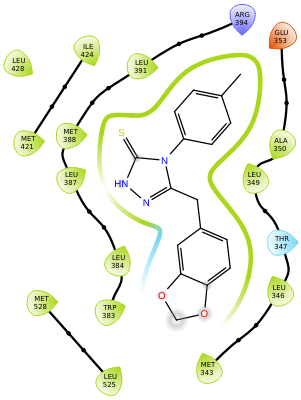


**Fig. S27** 2D docking pose of **6a**  **Fig. S28** 2D docking pose of **6b**


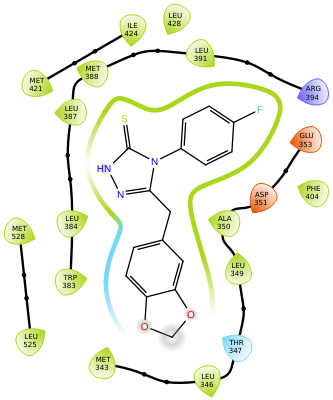

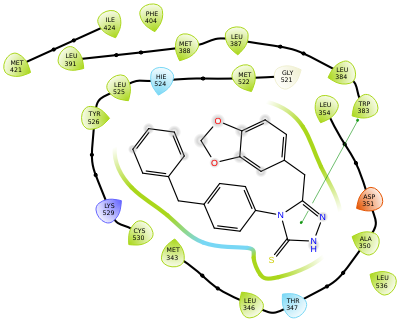


**Fig. S29** 2D docking pose of **6c Fig. S30** 2D docking pose of **6d**


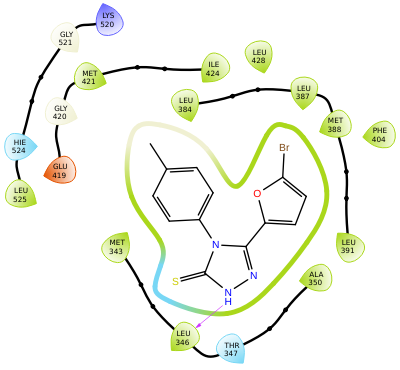

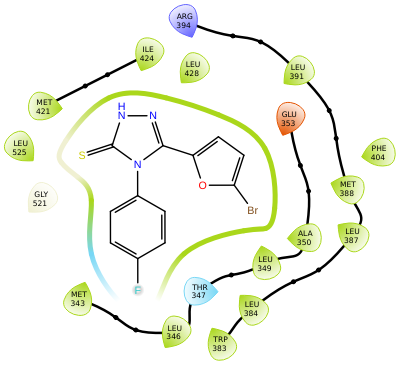


**Fig. S31** 2D docking pose of **12a Fig. S32** 2D docking pose of **12b**


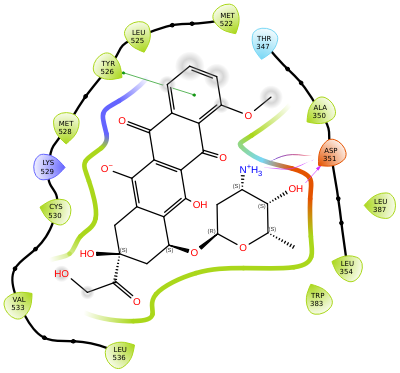


**Fig. S33** 2D docking pose of **Doxorubicin**

**3D docking poses**


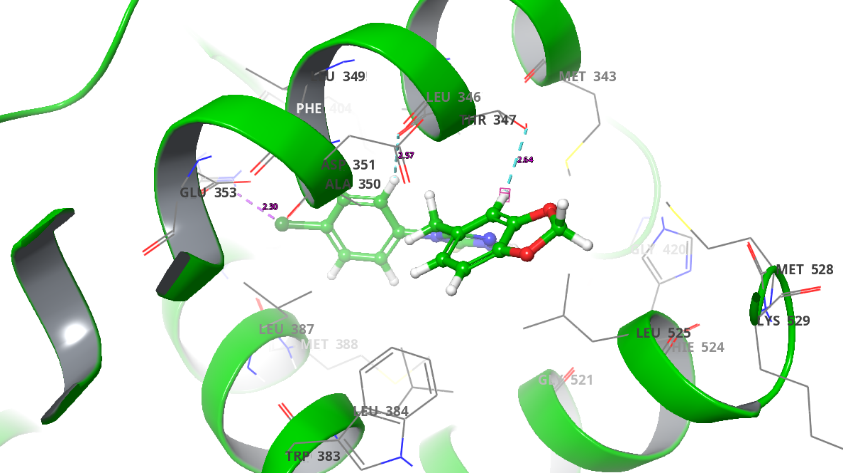


**Fig. S34** 3D docking pose of **6a**


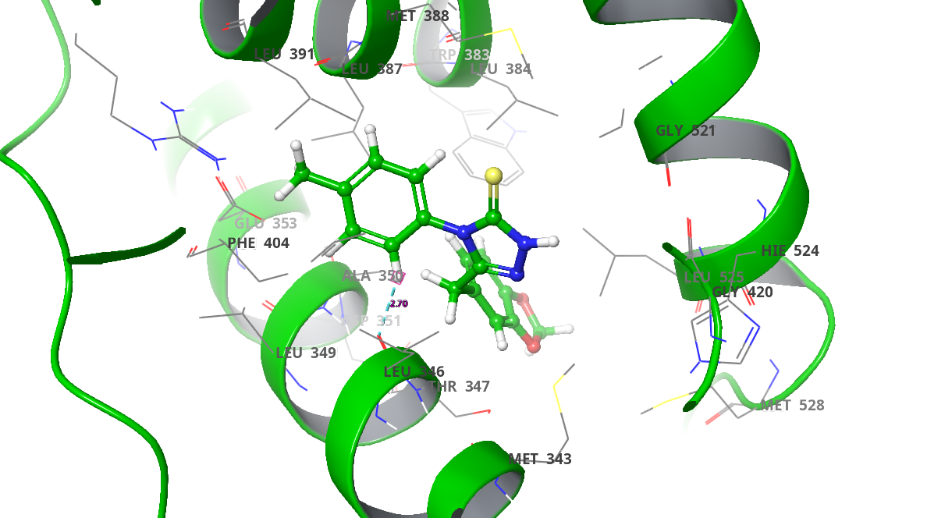


**Fig. S35** 3D docking pose of **6b**


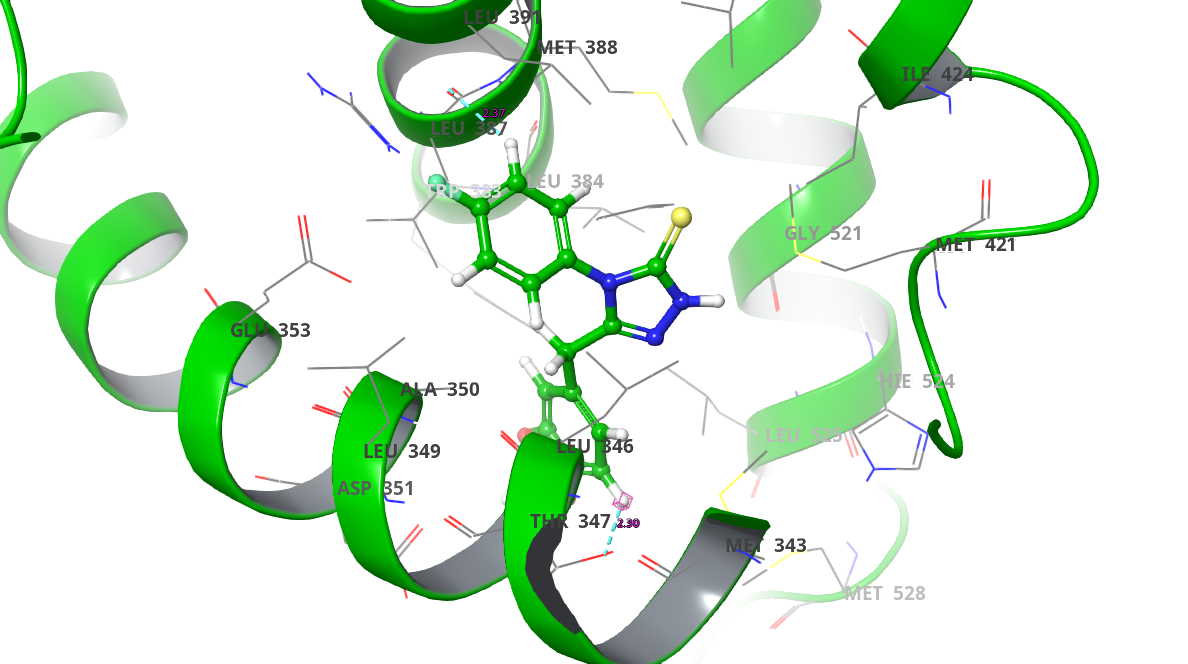


**Fig. S36** 3D docking pose of **6c**


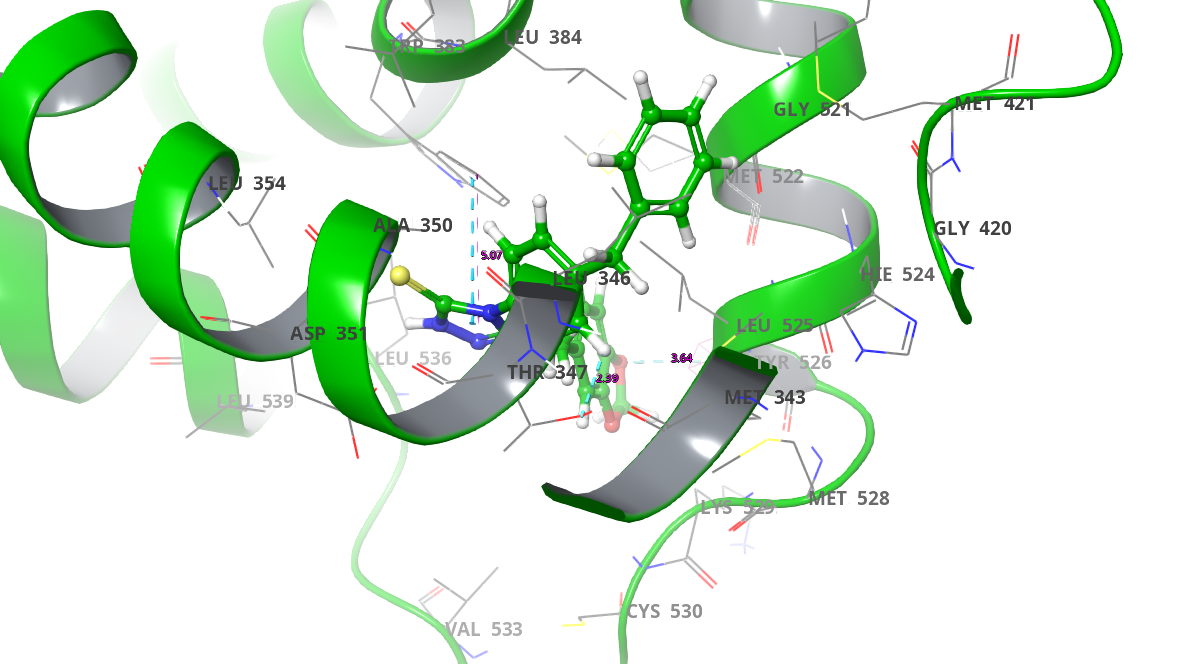


**Fig. S37** 3D docking pose of **6d**


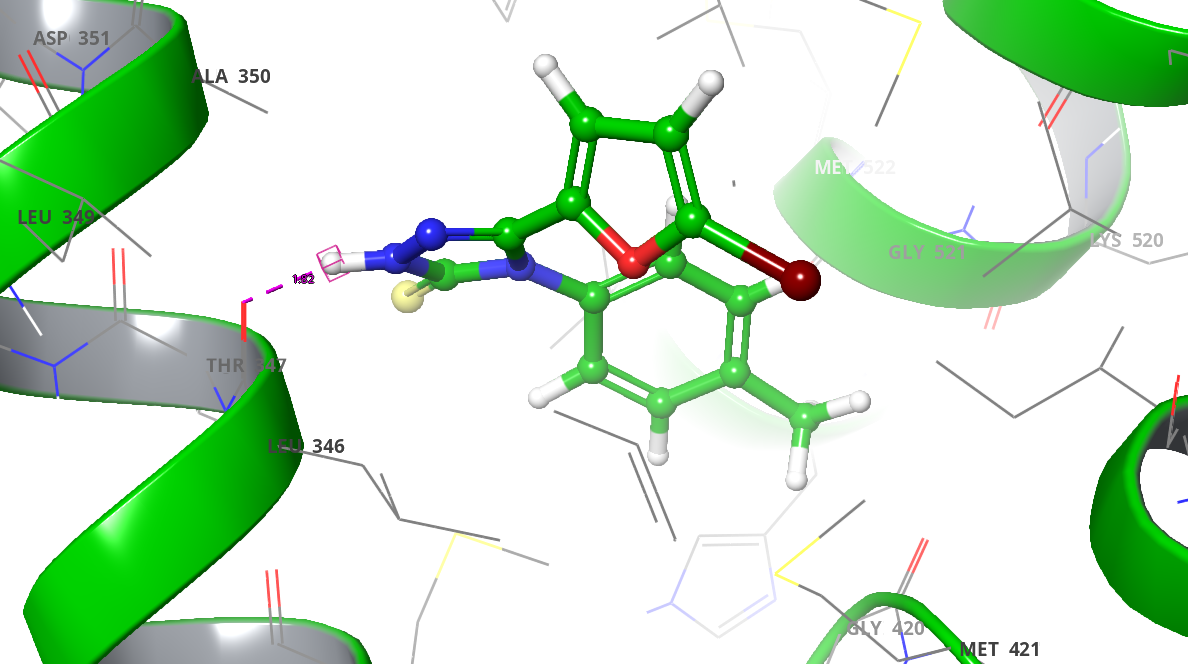


**Fig. S38** 3D docking pose of **12a**


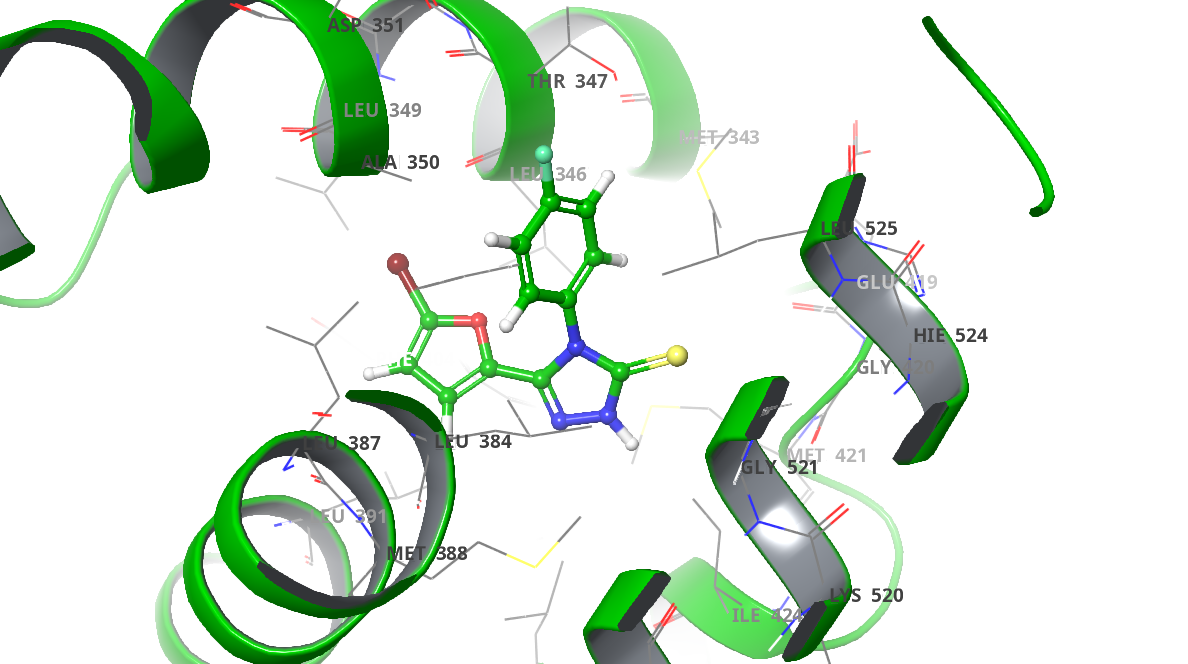


**Fig. S39** 3D docking pose of **12b**


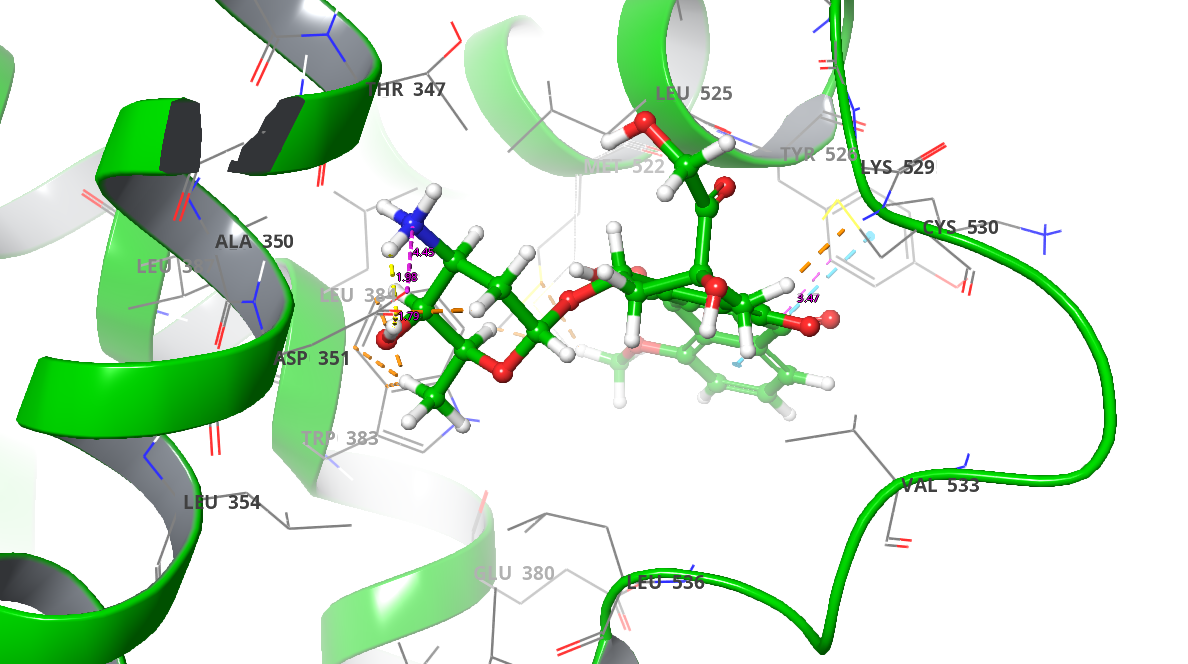


**Fig. S40** 3D docking pose of **Doxorubicin**
